# Vitality as a measure of animal welfare during purse seine pumping related crowding of Atlantic mackerel (*Scomber scrombrus*)

**DOI:** 10.1038/s41598-022-26373-x

**Published:** 2022-12-19

**Authors:** Neil Anders, Sigurd Hannaas, Jostein Saltskår, Erik Schuster, Maria Tenningen, Bjørn Totland, Aud Vold, Jan Tore Øvredal, Mike Breen

**Affiliations:** Institute of Marine Research (IMR), Fish Capture Division, Nordnes. NO-5817, Nordnesgaten 50, P.O. Box 1870, 5005 Bergen, Norway

**Keywords:** Behavioural methods, Conservation biology, Animal behaviour, Animal physiology, Marine biology, Ichthyology

## Abstract

The impacts of wild capture fishing on animal welfare are poorly understood. During purse seine fishing for Atlantic mackerel (*Scomber scrombrus*), catches are crowded to high densities to facilitate pumping onboard. This study aimed to monitor fish welfare during crowding events in the Norwegian purse seine fishery, and to identify relevant drivers. We first correlated a suite of neuro-endocrine, physiological and physical stress responses (integrated into a single measure of welfare using multivariate analysis) to the behavioural vitality of individual mackerel in controlled crowding trials in aquaculture cages. Vitality was found to be a useful measure of welfare. We then assessed individual fish vitality onboard a commercial purse seiner. Catch welfare, measured using vitality, was observed to be negatively impacted during pumping related crowding. Larger catches and longer crowding exposure times resulted in greater negative impacts. Vitality was not significantly impacted by crowding density or dissolved oxygen concentrations inside the net, although methodological limitations limited accurate measurement of these parameters. Blood lactate levels correlated negatively with vitality, suggesting that high-intensity anaerobic locomotory activity was associated with the reduction in welfare. Based on these findings, catch welfare could be improved by targeting smaller schools to minimise crowding exposure times.

## Introduction

Despite being the arena in which most human-fish interactions occur^1^, the welfare of fish during wild capture is an understudied phenomenon^2^. This stands in contrast to the vast numbers of individuals involved and a growing body of literature that demonstrates wild capture can be stressful for fish^3^. As reducing stress during capture could lead to ethical, sustainability and product quality improvements^4^, catch welfare should be a topic of interest to various stakeholders. The lack of information on the impacts of wild capture is therefore likely related somewhat to the difficulties in monitoring animal welfare in the dynamic and challenging conditions onboard commercial fishing vessels.

There are different approaches to what animal welfare constitutes^1^. In this study, we use a functional definition of welfare, which posits that an animal has good welfare when its biological systems can cope with environmental stressors appropriately and within their capacity^1^. This implies that welfare status can be assessed objectively by measuring biological systems and the consequences of stress. Diggles et al.^5^ advocate for the use of functional definitions when assessing welfare in wild capture fisheries, whilst recognising that such a definition does not necessarily depend upon consciousness or the ability to suffer in fish.

Exposure to acute stressors results in the activation of a wide range of regulatory mechanisms, which have evolved to achieve an appropriate statis so that any associated biological cost (also called “allostatic load”) can be minimized^6^. Consequently, quantifying changes to these regulatory mechanisms and their consequences should allow welfare inferences to be drawn. The response of regulatory mechanisms to stress is typically exhibited on three levels of biological organization: neuro-endocrine (primary responses), physiological (secondary responses) and at the whole-animal level (tertiary responses such as behaviour^1^). Measurement of one or more indicators at these different levels is therefore common in the assessment of animal welfare^7,8^. To be informative, a stress indicator should be quantifiable in an objective way, correlate to the amount of stress received, respond rapidly and be practical to measure^9,10^.

Accurate quantification and interpretation of stress indicators can be difficult to achieve^11^. For instance, some neuro-endocrine responses are detectable within seconds, meaning they are highly susceptible to handling effects^12^. Physiological responses often need to be quantified rapidly or adequately stored to be considered reliable^13,14^. Authors have also questioned how closely they reflect true welfare state of the animal^15,16^. Although popular and easy to measure^17^, interpreting behavioural measures of welfare is also difficult unless their motivational and neuro-endocrine/physiological basis is properly understood^18^. Considering these various limitations, welfare inferences are most robust when they are based on a variety of stress indicators at different levels of biological organization^19^.

It can therefore be stated that welfare status of an animal is a multi-faceted concept that is best determined when multiple metrics are considered. This suggests that assessment of welfare should be based on the integration of various stress responses. Multivariate techniques such as principal component analysis (PCA) are advantageous in this regard as they allow the objective weighting of various stress responses into a single “welfare index”, depending on how metrics relate to one another^20–23^. However, the complexity and cost of routinely measuring several stress metrics can be prohibitive, especially in field environments where access to analytical equipment may not be readily available. Correlating integrated welfare scores to more easily measured metrics can help to overcome this limitation^24–27^.

Vitality is a measure of how alive an animal is. It can be assessed objectively by determining the presence or absence of a suite of species-specific reflex and injury criteria in individual animals during observation/handling^28^. Due to this, it is relatively simple and inexpensive to conduct and requires little to no specialized equipment. Previous work has shown that it correlates to stressor intensity and duration, and mortality outcomes, in a range of aquatic taxa^29–35^. As such, vitality represents a potentially useful tool when assessing the welfare of animals, and one that can be applied in field settings where other neuro-endocrine or physiological methods may not be practical.

Purse seine is a globally important wild fish capture method^36^. In European waters, this gear type is used primarily to target small pelagic schooling species. The mode of capture involves encircling schooling fish with a small-meshed net, which is then closed from below^37^. Once the school is trapped, net volume is progressively reduced to concentrate the catch alongside the vessel so that it can be pumped aboard. Catches are typically in the order of several hundreds of tons^38^. The reduction in net volume results in the catch becoming crowded, which can become extreme at the end of the capture process during the time the fish are pumped aboard. Depending on catch size, pumping may take up to an hour or more to complete^39^. The exact densities experienced by fish during this time are difficult to measure due to a lack of appropriate monitoring methodologies, but could exceed 200 kg/m^3^^40^. The high biomass to water ratio means that reductions in ambient oxygen levels can also occur^40^. Consequently, crowding has been shown to result in a wide range of stress responses for a variety of small pelagic species during controlled experiments^9,41–46^ and at-sea observations^47,48^.

Atlantic mackerel (*Scomber scrombrus*) support economically important purse seine fisheries throughout European waters^49^. However, this species is particularly sensitive to the type of stress associated with purse seine capture; large scale mortality can occur if mackerel are crowded and subsequently released^50,51^ and the physiological challenge associated with this stressor can reduce the quality of the flesh^44,52^. In previous studies, we have described a range of behavioural and physiological metrics that are responsive to crowding stress in this species^9,41,42,44,53^ with a view of developing ways in which welfare can be assessed. This work to date has been laboratory or mesocosm-based using simulated crowding, and, as such, it has been questioned how well these experiments reflect the scale and stressors present in large commercial catches^41^. There is therefore a need to assess the welfare of mackerel during real capture events.

In this study, we assessed the welfare of mackerel and its drivers during pumping related crowding events in the Norwegian purse seine fishery, with a view to identifying ways in which welfare could be improved. Considering the logistical challenges of working onboard commercial fishing vessels, welfare was determined via vitality assessment as this can be easily achieved in the field without specialized equipment. Recognizing that welfare inferences drawn from a single stress metric such as vitality are questionable, we first correlated vitality to a suite of neuro-endocrine, physiological and physical stress metrics during a series of controlled crowding trials in sea cages. These metrics were integrated into a single measure of welfare using multivariate analysis. Specifically, we aimed to address the following research questions:

(i) Is vitality a useful measure of crowding stress in mackerel?
(ii) Does vitality correlate to welfare during crowding stress?
(iii) Is mackerel vitality affected by crowding during commercial pumping events in the fishery?
(iv) What drivers affect vitality, and hence welfare status, during pumping-related crowding events in commercial catches in the fishery?

## Methods

### Crowding trials in sea cages

The crowding trials described herein have been previously detailed in Tveit et al.^9^ and Anders et al.^41^ in which it was demonstrated that crowding of mackerel can result in physiological stress, a skin colour change towards blue, skin injuries, loss of allometric condition and delayed mortality events. We therefore detail only the pertinent details here. All procedures described were authorized by the Norwegian animal welfare authority (Mattilsynet, Licence ID: 19238), were conducted in accordance with relevant regulations and comply with ARRIVE guidelines^54^ where applicable.

#### Fish capture and simulating crowding

Feed pellets were used to benignly attract wild Atlantic mackerel into an offshore 1728 m^3^ aquaculture net cage at the Austevoll Research Facility (60° N) in the summers of 2018 and 2019. Inside the cage were two smaller experimental cages of 149.17 m^3^ in which the crowding trials took place. For these, ~ 150 fish were enticed to swim into the experimental cages using feed pellets and then given at least 2 days to acclimatize before beginning the trials.

A total of eight crowding trials were completed, in which we exposed groups of mackerel to crowding by lifting the experimental cages vertically in the water by hand to reduce the available swimming space. Fish were then held at a constant density for a given time (Table 1), before releasing the cage and allowing it to sink and return to its unrestricted volume. Cage volume was not changed for control trials. Our aim was to induce a range of different stress levels by manipulation of stressor intensity (the crowding density) and its duration (crowding exposure)^55^. The densities and durations broadly simulated the range fish are likely to experience during capture and pumping events in the Norwegian purse seine fishery^39,53,56^. Density was estimated after crowding based on the biomass of fish and the geometric shape of the cage, under the assumption that fish adopted a density dependent on cage volume (refer to Anders et al.^41^ for full details). During treatment, we monitored water temperature (°C) and dissolved oxygen concentrations (DO_2_) in the cages using a SAIV CTD (Model: SD204) fitted with a RINKO III oxygen sensor (JFE Advantach Co., Ltd). The CTDO was placed in the approximate centre of the school of fish.

**Table 1:** Pertinent details of cage trials used to assess the impact of crowding on the welfare of Atlantic mackerel (Scomber scrombrus).

Trial name	Dates (from treatment–trial end)	Treatment duration (decimal hours)	Estimated during-treatment density (kg/m^3^)	No. of fish exposed to treatment	Mean (± 95% confidence interval) temperature (°C)	Mean (± 95% confidence interval) oxygen concentration (mg/L)	Mortality proportion (95% confidence bounds*)
Control 1	21/05/19 – 21/05/19	1.87	0.76	149	11.608 ± 0.007	10.247 ± 0.004	Survival not monitored
Control 2	28/05/19 – 06/06/19	0.68	0.59	116	10.991 ± 0.003	Data missing	0.000 (0.000, 0.038)
High & Prolonged 1	22/05/19 – 22/05/19	1.13	Data missing**	78	10.799 ± 0.001	9.339 ± 0.012	Survival not monitored
Low	29/05/19 – 06/06/19	0.25	92.00	231	Data missing	Data missing	0.000 (0.000, 0.018)
High & Prolonged 2	06/06/19 – 06/06/19	1.15	182.75	91	13.880 ± 0.024	9.080 ± 0.016	Survival not monitored
Control 3	21/08/19 – 17/09/19	0.75	0.76	175	16.425 ± 0.001	8.386 ± 0.001	0.000 (0.000, 0.024)
Moderate	22/08/19 – 11/09/19	0.22	146.21	131	16.067 ± 0.002	6.182 ± 0.014	0.026 (0.009, 0.074)
High	28/08/19 – 17/09/19	0.25	179.87	150	17.883 ± 0.002	6.781 ± 0.028	0.305 (0.233, 0.389)

*Calculated from Wilson score intervals.

**Density assumed to be similar to the “High & Prolonged 2” trial based on qualitative observations.

#### Measuring stress responses

For animal welfare reasons, cages exposed to "High & Prolonged” crowding (Table 1) were removed from the trials immediately after treatment. Otherwise, cages were monitored twice daily for moribund or dead individuals for up to 27 days^41^. To characterize stress responses, we collected fish from cages prior to, during and after crowding (at 2 h, 24 h, and at the end of the trial). We aimed to collect as many fish as possible during crowding (mean number of fish sampled ± 95% confidence interval [CI] was: 8 ± 1.13) and five individuals in the other collection periods. The time at which the first fish was removed for sampling was considered as the start of treatment. During crowding or for moribund individuals, we collected fish using a landing net. Otherwise, we encouraged feeding behaviour with pellets and caught fish using a barbless hook and handline. Where logistically possible, all collected fish were assessed for vitality (see details below), euthanized by a percussive blow to the head, photographed to determine stress related skin colour change^9^ and had blood collected from the caudal vasculature to characterize physiological status^41^. From collection to blood sampling typically took < 1 min. Fulton’s condition factor^57^ (K) was calculated as follows: K = 100 × total weight (g)/fork length (cm)^3^. Mean (± 95% CI) uncrowded fish fork length, weight and condition factor was 38.36 ± 0.37 cm and 738 ± 24 g and 1.30 ± 0.02 g cm^−3^, respectively^41^. Haematocrit, plasma pH, potassium ions (K^+^), sodium ions (Na^+^), chloride ions (Cl^−^), glucose, lactate, cortisol and osmolality were quantified from the blood samples (refer to Anders et al*.*^44^ for detail of analytical techniques). The photographs were digitally analysed, with b* (blue-yellow component) being quantified according to CIELAB colour space (refer to Tveit et al*.*^9^ for further detail).

#### Measuring vitality

Upon collection, fish were immediately assessed for vitality. The vitality metrics (Table 2) were based on Davis et al.^28^. One of two different vitality procedures were performed on alternate fish: (1) fish placed in a 70-l seawater tank, assessed for “free swimming” vitality metrics, then assessed for vitality “while handling”; or (2) just the handling vitality assessment. The overall vitality score [VS] for an individual fish was calculated as the sum of scores (1 = present; 0.5 = weak or uncertain; and 0 = absent) from up to eight separate vitality metrics [*v*_*i*_] divided by the total number of vitality metrics [*V*] successfully recorded for that fish:

**Table 2:** Criteria used to assess the vitality of Atlantic mackerel (*Scomber scrombrus*) during crowding trials.

Procedure	Metric	Methodology	Positive response
Free swimming observations	Evasion 1	Fish transferred into observation tank	A "startle" response, or swims around tank seeking "escape"
	Orientation/self-righting	Fish transferred into observation tank	Self-orientation (dorsal side up) ≤ 5 secs of transfer
	Head complex	Fish transferred into observation tank	Coordinated and regular movement of mouth and operaculae—indicative of normal respiration (> 1 per 10 secs)
	Evasion 2	Observer's hand, in water, approaches fish from side; in preparation for "caudal reflex” test (see below)	A "startle" response, or swims around tank seeking "escape"
	Caudal reflex	Observer touches, or attempts to hold, caudal fin	Immediate (< 1 secs) attempt to swim away from physical contact
Observations while handling	Body flex 1—restrained	Fish firmly held in clenched hand, with thumb and fore-finger just posterior of operculae (NB: test starts in water, as fish is removed from tank)	Tail musculature flexes within 3 secs of test initiation
	Vestibula-ocular response	Observer (while holding fish as described above) rotates fish on the longitudinal axis	Eyes hold steady, with respect to horizonal
	Mouth closure	Observer (while holding fish as described above) uses finger to open fish's mouth	Mouth opening action is resisted within 3 secs of test initiation. May also respond with a gaping motion of the mouth and operculae and/or flexing the tail musculature
	Body flex 2—flat surface	Fish is laid, unrestrained, on a flat surface	Tail musculature flexes within 3 secs of test initiation

$$VS = ~{\raise0.7ex\hbox{${\sum\nolimits_{{i = 1}}^{V} {v_{i} } }$} \!\mathord{\left/ {\vphantom {{\sum\nolimits_{{i = 1}}^{V} {v_{i} } } V}}\right.\kern-\nulldelimiterspace} \!\lower0.7ex\hbox{$V$}}$$

We pooled VS of fish from the two assessment procedures in subsequent analysis because linear regression indicated the two were highly correlated (d.f. = 142, *F* = 391.1, *p* =  < 0.001; R^2^ = 0.89, Supplementary Table S1). The duration of the vitality assessment was on average (mean ± 95% CI) 74.3 ± 5.8 s (n = 184) and the additional in-water assessment procedure did not significantly influence any of the blood physiology parameters^41^.

To determine what influenced VS in the cages, we modelled the relationship between “exposure time” (continuous) and “DO_2_” (continuous) for each “trial” (categorical, refer to Table 1 for levels). “DO_2_” was determined from backwards looking moving averages of the oxygen conditions inside the cage. We averaged DO_2_ concentrations during the 1, 3 and 5 min periods prior to vitality sampling. All fish in the “Control 1” trial had full vitality; this level was therefore removed from modelling to prevent separation issues during model fitting^58^. Three candidate model sets (one each for the three DO_2_ time averages) were generated consisting of various combinations of the covariates, and the most parsimonious model in each set selected according to AICc (Akaike Information Criteria corrected for small sample size) and AICw (normalized Akaike weights). All candidate models contained at least “exposure time” and “trial” to reflect the experimental setup and had a beta error structure (logit link). Our VS were on the interval of [0,1]. To allow beta regression, the VS were transformed to the open interval of (0,1) in accordance with Smithson and Verkuilen^59^.

#### Generating a welfare score

Functional welfare is the holistic expression of a variety of physiological and neuro-endocrine responses^1^. After Turnbull et al.^21^, we therefore combined our physiological, injury, allometric condition and skin colour metrics of mackerel using a Hill and Smith principal component analysis (PCA). By maximizing the sum of the squared correlations for quantitative variables (i.e. the physiological, allometric condition and skin colour metrics) or correlation ratios for categorical variables (i.e. injury status), separate vectors (principal components) that best approximate the data is obtained with single scores upon these vectors for individual fish^60^. Prior to conducting the PCA, case wise deletion of any fish with missing values for any of the metrics was performed and variables were centred and normalized. As we were unable to collect skin colour photographs during the “High & Prolonged 2” trial due to logistic reasons, this trial was removed from the PCA.

**Figure 1:**
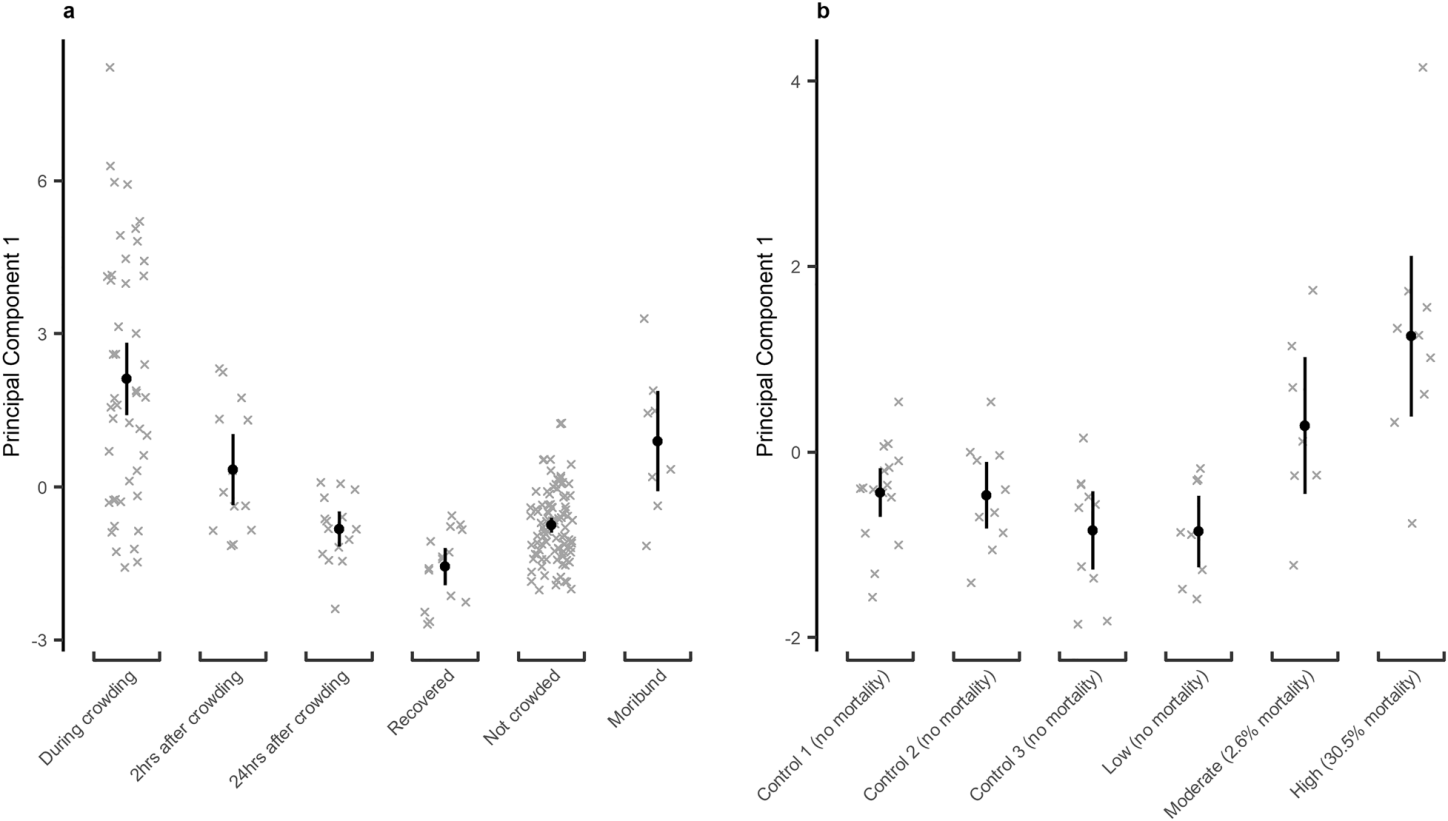
The relationship between treatment of Atlantic mackerel (*Scomber scrombrus*) in crowding trials and Principal Component 1 from a Hill and Smith principal component analysis. (**a**) The relationship with fish sampled at various time points during all trials. (**b**) The relationship with overall mortality rates for the different trials for fish sampled during treatment periods. Points indicate model derived mean values with 95% confidence intervals as whiskers. The underlying dataset is indicated as crosses.

The PCA generated three vectors with eigenvalues > 1 (and therefore worthy of further investigation^60^) that together explained ~ 64% of the data variance (principal component 1 [PC1]: 29.3%; principal component 2 [PC2]: 17.1%; principal component 3 [PC3]: 16.2%). PC1 was loaded primarily by Na^+^, lactate, osmolality, pH, cortisol (positive loading) and CIELAB b* skin colour (negative loading, Table 3). These loadings are consistent with our previous work on crowded mackerel^41,44^. Generalized least squared (GLS) regression indicated a significant (Wald *F* testing: df = 5, *F* = 20.38, *p* < 0.001, Supplementary Table S2) relationship between PC1 scores and how mackerel were treated during trials. PC1 scores obtained during crowding were also different between trials (df = 5, *F* = 5.21, *p* < 0.001, Supplementary Table S3). Significantly higher PC1 scores were obtained during crowding than for fish which never experienced crowding, and scores tended to reduce post-crowding (Fig. 1a). Moribund fish also had significantly higher PC1 scores than non-crowded fish (Fig. 1a). PC1 scores from during crowding increased with increasing rates of mortality and were generally higher than for trials in which mortalities did not occur (Fig. 1b). The PC1 scores therefore represented a single measure of mackerel welfare that increased with worsening welfare. To aid in interpretation, we transformed the PC1 values into a “welfare score” by multiplying by − 1. Consequently, a lower “welfare score” represents a worse welfare state.

**Table 3:** Loadings of various Atlantic mackerel (*Scomber scrombrus*) stress responses in a Hill & Smith principal component analysis.

Welfare metric	Loading on principal component 1	Loading on principal component 2	Loading on principal component 3
Haematocrit	0.156	0.334	− 0.284
pH	− 0.267	0.531	0.061
K^+^	0.119	− 0.483	0.112
Na^+^	0.490	− 0.042	− 0.120
Cl^-^	0.157	0.407	0.349
Glucose	0.088	0.341	− 0.299
Lactate	0.475	0.036	0.072
Cortisol	0.259	0.200	0.294
Osmolality	0.469	0.006	0.022
CIELAB b*	− 0.307	− 0.057	− 0.061
Condition factor	0.103	− 0.164	− 0.507
Injured	− 0.063	− 0.501	2.119
Not injured	0.005	0.036	− 0.153

Only principal components with eigenvalues > 1 are included.

With regards to PC2 and PC3, both vectors were dominated by high loadings on injuries, and in the case of PC2 by positive loading on pH and negative loading on K^+^ (Table 3). Both vectors were related significantly to mackerel treatment in the cage trials (GLS modelling with Wald *F* testing—PC2: df = 5, *F* = 5.18, *p* < 0.001; PC3: df = 5, *F* = 83.55, *p* < 0.001, Supplementary Tables S4, S5) with scores for moribund fish being substantially lower than other individuals for PC2 and significantly higher for PC3 (Supplementary Fig. S1). Elevated levels of K^+^ and injuries characterized moribund fish profiles in the cage trials, where injuries took ~ 2 days to develop^41^. Taken together, these finding suggest PC2 and PC3 are best suited to describing the welfare of moribund fish with skin injuries, with little information with regards to welfare during crowding. These vectors therefore warranted no further attention with regards to the objectives of this study.

#### Establishing the relationship between welfare and vitality

We examined the welfare (i.e. the PCA generated welfare scores)–VS relationship using a linear mixed model (Gaussian error structure and identify link). To account for potential heterogeneity and to establish the population level relationship^58^, we included a random slope term for the welfare–VS relationship for each trial, with a fixed intercept (to reflect that fish all with a VS = 0 must, by definition, have the same level of welfare).

### Fishery observations

#### Measuring vitality and confirming a stress response

We collected vitality data from 3 separate trips onboard the same purse seine vessel, operating commercially in the Atlantic mackerel fishery in the Norwegian Sea during the autumns of 2019 and 2020. The vessel (“FV Fiskebas”) was a typical modern offshore Norwegian purse seiner: 55 m LOA, operating a 746 × 212 m (length × depth) net. Once a suitable school was located, the vessel encircled it with the purse seine, closed it from below and then hauled the net until the catch was densely crowded alongside. A SeaQuest (18″ diameter) impellor pump was used to transfer the catch aboard.

Fish were randomly collected one at a time using a landing net as they passed over the dewatering unit following pumping, assessed for VS using the full suite of metrics (both “free swimming” and “while handling”, Table 2) and then euthanized as described for the cage trials. Sampling continued until the vessel completed pumping. Vitality assessment onboard was faster than that achieved during the cage trials, with an average time (mean ± 95% CI) of 58.8 ± 2.6 s (n = 260, likely due to the improved experience of the assessor). Vitality assessment was conducted by the same assessor as during the cage trials. We recorded individual fish lengths and weights, and calculated Fulton’s condition factor, as described for the cage trials.

The physiological response of mackerel during the cage trials was characterized by increased lactate levels (Table 3 and Anders et al.^41^). We were therefore interested in confirming that a similar response was present during fishery events to give support to the use of vitality as a field measure of welfare. Accordingly, we collected blood from the caudal vasculature (using EDTA treated 5 ml syringes with 21G needles) immediately following euthanization. We quantified blood lactate levels from these samples using a Lactate Pro 2 (Arkray Inc., Kyoto, Japan) point-of-care device, which has been validated for measuring relative changes in lactate levels in Atlantic mackerel^44^. We fitted a generalised linear mixed model (gamma error structure with log link) to the lactate data, with VS as a fixed effect and a random intercept term for each observed catch nested with within trip^58^.

#### Monitoring potential drivers of vitality

As potential drivers of vitality during pumping, we considered: (1) crowding exposure time; (2) crowding density; (3) catch size; (4) the depth at which fish were pumped from the net; and (5) DO_2_. As the measure of exposure time, we recorded the duration from the start of pumping to the point of sampling for each fish. Although fish experience some degree of progressive crowding prior to this point as the net is hauled after setting^61^, the start of pumping represents a convenient reference point to assess relative changes in vitality. It was not feasible to measure actual crowding densities due to a lack of suitable monitoring technology. We therefore recorded the rate at which the catch was pumped onboard (tonnes/h) as its proxy, under the assumption that a more densely crowded catch would have a faster pumping rate. Although fish are continually removed during pumping, densities remain broadly stable as net volume is adjusted by fishers to maintain an efficient pumping rate. We determined catch size from water displacement and ullage measurements in the onboard refrigerated seawater storage tanks. To monitor DO_2_, we deployed a RINKO ID oxygen, temperature and depth logger (JFE Advantech, 2021) into the crowded catch. The logger was attached to the outside of the pump head inside a protective housing. In addition, a similar protective housing containing another RINKO ID logger was suspended at a depth of ~ 5 m below a float and allowed to drift inside the net. This was deployed into the net prior to pumping and remained there until just before pumping commenced. In this way, we gathered environmental data at various locations in the net prior to and during the pumping related crowding. We determined the DO_2_ conditions experienced by fish in the 1, 3 and 5 min prior to vitality assessment by using backwards moving averages as for the cage trials, but with an offset of 30 s (to account for the approximate time the animal was in the pump system prior to sampling^39^).

#### Determining the drivers of vitality

Data exploration indicated a high degree of collinearity between catch size and all other candidate continuous predictor variables (Supplementary Figs. S2–S4). This collinearity was too high (Variance Inflation Factor [VIF] = 7.75) to justify inclusion of this term in a global model^62^. However, without catch size, the collinearity between the other candidate predictors was acceptable (VIF < 1.7 in all cases). Consequently, we fitted two model sets to explain the VS obtained at sea. The first considered just “catch size” (continuous) as a predictor variable. The second considered “exposure time” (continuous), “pumping rate” (continuous), “DO_2_” (continuous) and “pump depth” (categorical: “ < 5 m depth” or “ > 5 m depth”). For the second set, three groups of candidate models (one each for the three DO_2_ time averages) were constructed using various combinations of the predictors (but always containing at least “exposure time” and “pumping rate”). Models were then ranked according to AICc and AICw. Where there was equal support (ΔAICc < 2) for top ranked models, we conducted model averaging^63^. For this, a weighted average of each parameter estimate contained within the top competing models was obtained using the “zero method”^64^. All models were fitted with beta error structures (logit link), included a random intercept term for each observed catch nested within trip, and had VS transformed as for the cage trials. All continuous predictors were scaled according to their respective means and SD.

All statistical analyses were undertaken in R (version 4.1.1). Models were checked for violation of assumptions using residual plots (generated using the DHARMa package in the case of mixed models^65^). Residual heteroskedasticity was incorporated into models using variance structures^58,66^. Significance of model terms was determined using likelihood ratio testing (LRT) unless otherwise stated.

## Results

### Crowding trials

**Figure 2:**
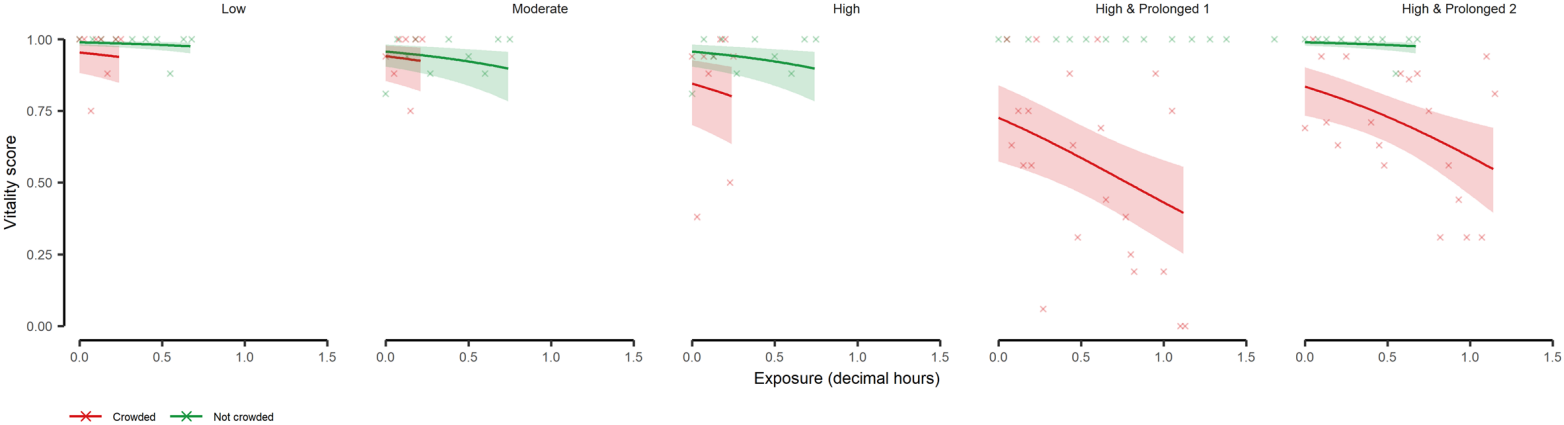
Atlantic mackerel (*Scomber scombrus*) vitality during exposure to crowding stress. Groups of wild-caught mackerel were crowded at different densities and durations in sea cages. No model was fitted to the control treatment for the “High & Prolonged 1” trial because all fish had full vitality (Vitality score = 1). The model derived relationship is indicated by the solid lines with the shaded areas indicating the associated 95% confidence intervals. The underlying dataset is indicated as crosses.

The vitality scores (VS) from the cage trials were most parsimoniously described by main effects of “exposure time” and “trial” (refer to Table 4 for ranking of the model set considering the DO_2_ 1 min averages, and Supplementary Tables S14 and S15 for the 3 and 5 min DO_2_ sets). The vitality of mackerel in the crowding trials decreased with increasing exposure time (df = 1, LRT = 8.872, *p* ≤ 0.01) and depended strongly on the applied density (df = 6, LRT = 38.685, *p* < 0.001, Supplementary Table S6) (Fig. 2). Although there was a considerable variation between individual fish when crowded, higher densities generally resulted in greater vitality reductions with no appreciable change for non-crowded control fish (Fig. 2). Reductions in vitality due to crowding were rapidly evident, often by the start of sampling (i.e. exposure time = 0, Fig. 2).

**Table 4:** Ranking of candidate generalized linear mixed models (beta error structures with logit link) to explain the vitality of Atlantic mackerel (*Scomber scombrus*) during sea cage crowding trials.

Rank	Fixed effect variables	Log likelihood	ΔAICc	Weight
1	Exposure time + Trial	42.796	0.000	0.684
2	Exposure time + Trial + DO_2_ (1 min)	42.931	2.33	0.213
3	Exposure time + Trial + Exposure time × Trial	46.192	4.17	0.085
4	Exposure time + Trial + DO_2_ (1 min) + Exposure time × Trial	46.342	6.86	0.022

The “DO_2_ (1 min)” term indicates a moving average of dissolved oxygen conditions in the cage 1 min prior to vitality sampling. AICc refers to Akaike Information Criteria (corrected for small sample size) and “Weight” to normalised Akaike weights.

There was considerable support (as indicated by the ΔAICc) for the next best competing models, which additionally contained dissolved oxygen concentration (DO_2_, Tables 4, Tables S14, S15). However, the effect of DO_2_ in these models was always non-significant (1 min: df = 1, LRT = 0.269, *p* = 0.604; 3 min: df = 1, LRT = 0.297, *p* = 0.586; 5 min: df = 1, LRT = 0.245, *p* = 0.621).

**Figure 3:**
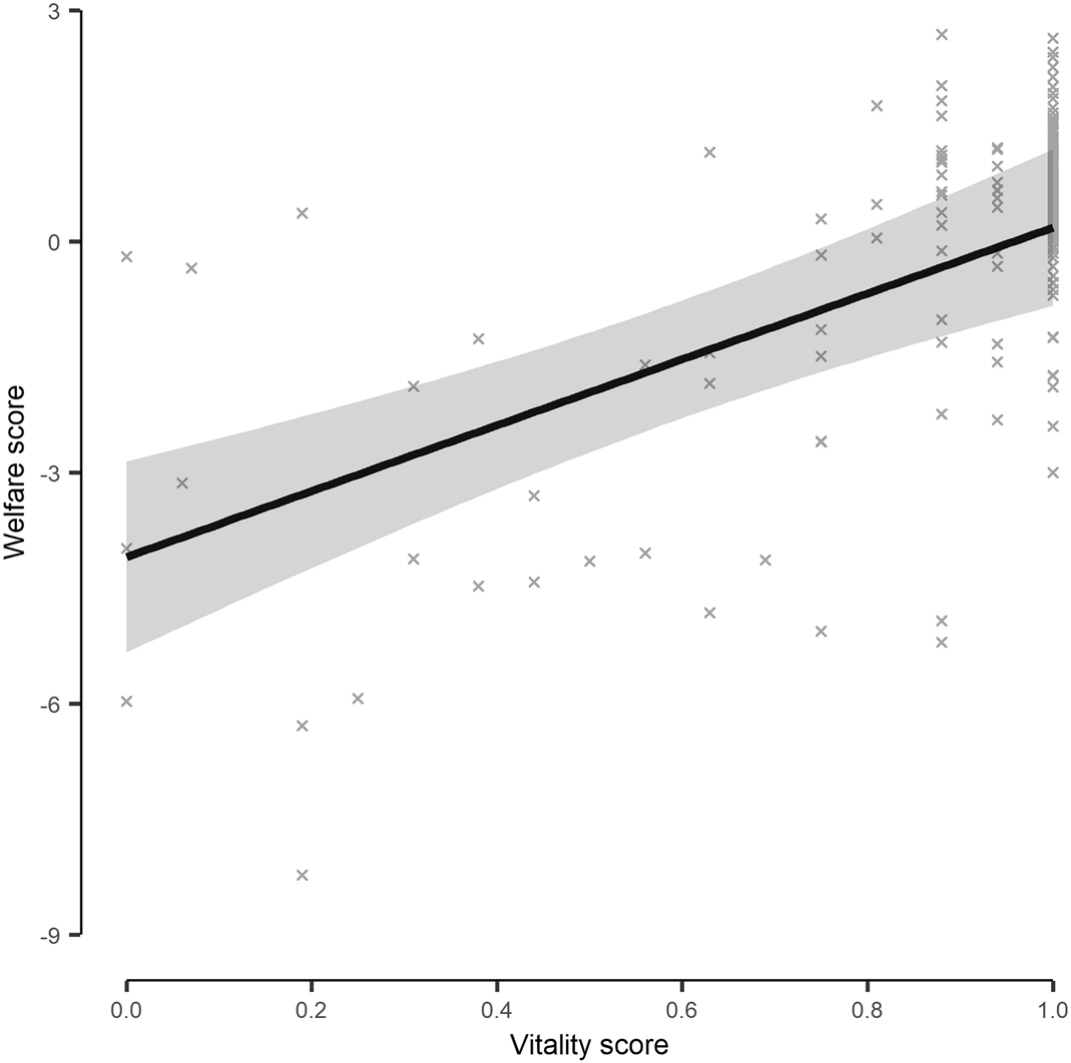
The model derived relationship between vitality scores and welfare scores for individual Atlantic mackerel (*Scomber scombrus*) during crowding stress cage trials. Welfare scores were based on physiological, physical and skin colour stress responses and combined in Principal Component 1 of a Hill & Smith principal component analysis. Higher welfare scores are indicative of worse welfare. The shaded area indicates the model derived 95% confidence interval, with the underlying data indicated as crosses.

VS was a statistically significant predictor of the PCA generated welfare score (df = 1, LRT = 14.90, *p* ≤ 0.001, Supplementary Table S7). Higher vitality scores were generally indicative of better welfare (Fig. 3).

### Fishery observations

We measured mackerel VS from 13 different pumped catches (Table 5). The number of fish we were able to assess per catch depended on the duration of pumping, with longer pumping allowing more fish to be sampled (Table 5). On average, 16 ± 8 (mean ± 95% confidence interval) fish per catch were assessed. Mean (± 95% CI) fork length, weight and condition factor was 33.95 ± 0.28 cm, 444 ± 11.85 g and 1.15 ± 0.01 g cm^−3^ respectively. The mean (± 95% CI) catch size was 151 ± 76 tonnes, with a mean pumping duration of 0.43 ± 0.20 h (equating to a mean pumping rate of 336 ± 33 tonnes/h).

**Table 5:** Pertinent details of purse seine catches in which the vitality of Atlantic mackerel (*Scomber scrombrus*) was assessed.

Trip	Catch code	Date	Catch size (tonnes)	Pumping duration (decimal hours)	Mean oxygen concentration (95% confidence interval bounds) in net prior to pumping (mg/L)	Mean oxygen concentration (95% confidence interval bounds) during pumping (mg/L)	Oxygen concentration minimum during pumping (mg/L)	Mean temperature (95% confidence interval bounds) (° C)	Range of vitality scores	Mean vitality score (95% confidence interval bounds)	Number of fish assessed for vitality
1	K02	19-09-19	220	0.67	8.96 (8.77—9.15)	7.37 (7.31–7.44)	4.31	10.18 (10.17–10.19)	0.220–1.000	0.729 (0.653–0.804)	29
	K04	22-09-19	400	1.15	10.2 (10.1—10.3)	6.51 (6.44–6.58)	2.59	10.96 (10.92–11.00)	0.000–1.000	0.751 (0.670–0.832)	30
	K11	29-09-19	40	0.13	10.1 (10.1–10.3)	9.23 (9.15–9.30)	6.00	12.31 (12.31–12.32)	0.060–1.000	0.695 (0.270–1.00)	4
	K12	29-09-19	30	0.14	9.98 (9.95–10.0)	9.60 (9.55–9.65)	7.58	12.21 (12.19–12.22)	0.330–0.830	0.657 (0.505–0.809)	6
	K13	29-09-19	190	0.56	9.69 (9.57–9.80)	7.96 (7.90–8.02)	4.41	12.19 (12.19–12.20)	0.280–1.000	0.783 (0.695–0.872)	19
2	L02	27-09-20	85.1	0.20	10.2 (9.90–10.4)	9.55 (9.45–9.64)	4.14	11.65 (11.64–11.66)	0.610–0.940	0.760 (0.582–0.838)	7
	L03	27-09-20	309.3	0.71	9.70 (9.45–9.95)	8.49 (8.44–8.54)	3–46	14.91 (14.79–15.03)	0.330–1.000	0.815 (0.748–0.882)	25
	L05	29-09-20	62.4	0.21	10.2 (10.2–10.3)	10.07 (10.03–10.12)	6.74	12.77 (12.70–12.84)	0.390–1.000	0.805 (0.658–0.952)	10
	L06	29-09-20	81.6	0.31	10.4 (10.3–10.5)	8.43 (8.32–8.55)	3.27	12.14 (12.14–12.15)	0.390–1.000	0.803 (0.703–0.903)	13
	L09	10-02-20	63.2	0.20	Not recorded	Not recorded	Not recorded	12.47 (12.45–12.50)	0.220–0.890	0.646 (0.478–0.813)	7
3	M01	16-10-20	44.2	0.13	10.1 (9.89–10.3)	9.44 (9.33–9.54)	6.35	11.30 (11.27–11.33)	0.500–0.940	0.769 (0.662–0.875)	7
	M02	16-10-20	33.6	0.09	11.5 (11.5–11.6)	11.04 (10.99–11.09)	7.60	10.69 (10.65–10.72)	0.780–0.890	0.856 (0.812–0.900)	5
	M04	16-10-20	406.5	1.08	10.2 (9.99–10.5)	7.18 (7.10–7.27)	2.18	11.46 (11.45–11.48)	0.000–0.940	0.642 (0.560–0.724)	43

**Figure 4:**
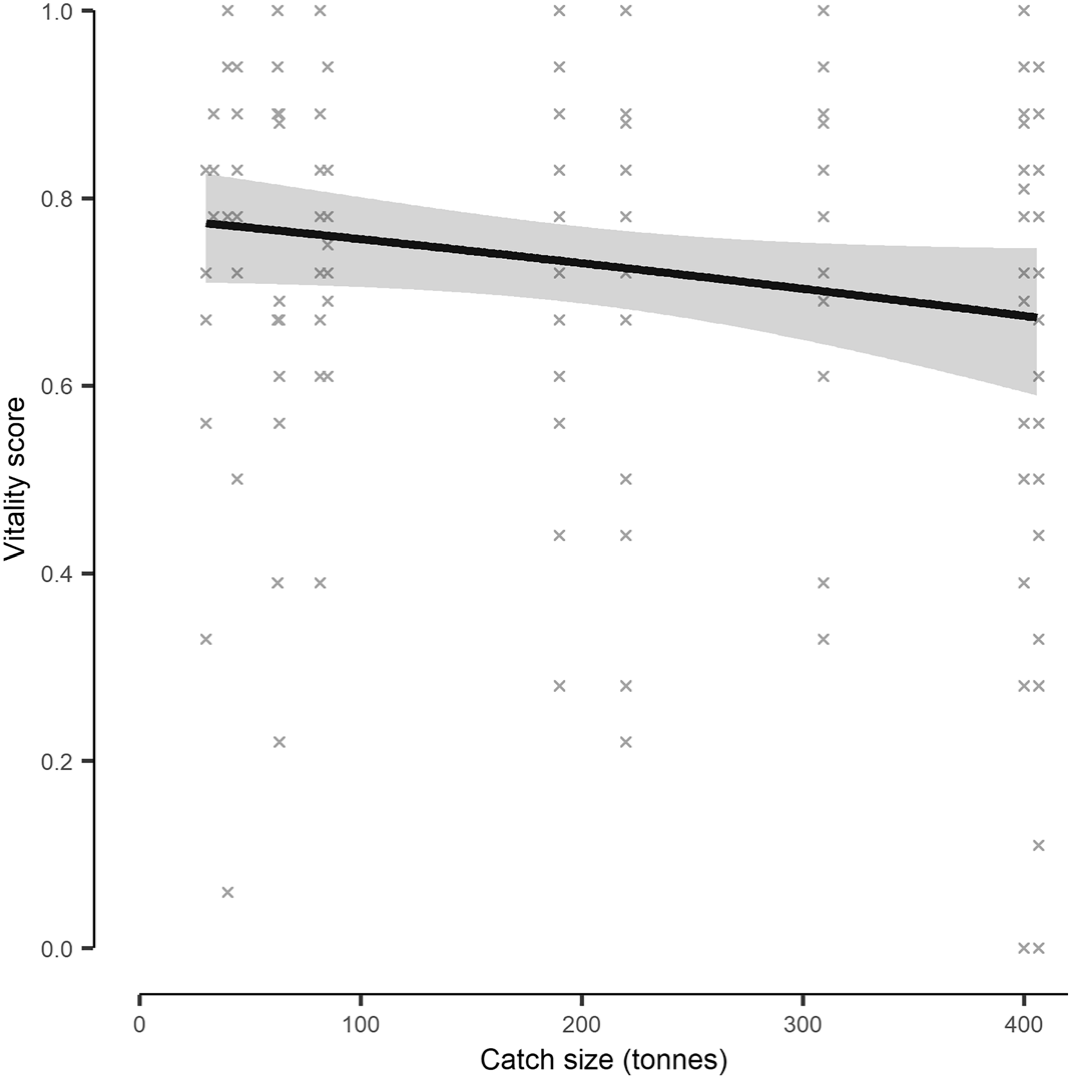
The model derived relationship between Atlantic mackerel (*Scomber scombrus*) vitality scores and catch size during crowding events onboard a commercial purse seine vessel. Fish were collected throughout crowding (after being pumped onboard) and assessed for behavioural vitality. The shaded area indicates the model derived 95% confidence interval, with the underlying data indicated as crosses.

Within individual pumping events, the vitality of mackerel was highly variable but tended to be lower in larger catches (Fig. 4). For each additional 100 tonne of catch, the catch size model (Supplementary Table S10) predicted mean vitality to reduce by ~ 3% (Fig. 4). This effect was, however, not statistically significant (df = 1, LRT = 2.659, *p* = 0.103). Vitality also tended to reduce towards the end of pumping when the net was mostly hauled in (as indicated by the relatively shallow pump depth) (Supplementary Fig. S5).

**Figure 5:**
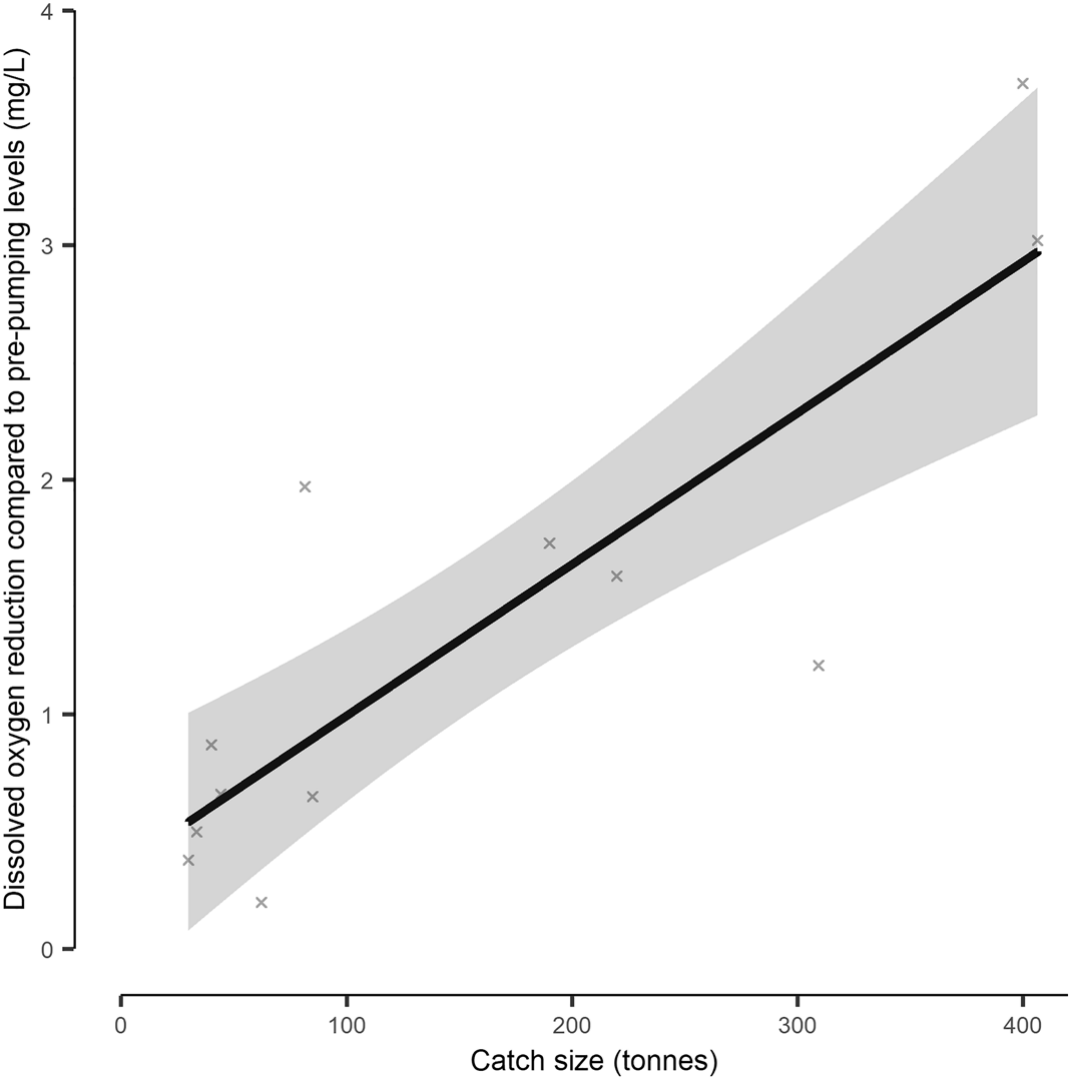
The model derived relationship between catch size and dissolved oxygen concentration reduction during pumping of Atlantic mackerel (*Scomber scrombrus*) purse seine catches. The reduction in oxygen is in comparison to pre-pumping levels. The shaded area indicates the model derived 95% confidence interval, with the underlying data indicated as crosses.

DO_2_ was variable throughout pumping (Supplementary Fig. S5). Larger catches (> 190 tonnes) typically had minima towards the beginning of pumping, with a gradual recovery over time. A second minima (usually smaller in magnitude) was also recorded in most large catches, associated with the end of the pumping phase when the last of the net was hauled in and the pump depth was substantially reduced. Smaller catches did not have such well-defined minima and always had shallow pumping depths (< 5 m). Across all catches, there was a mean (± 95% CI) reduction in DO_2_ of 1.37 ± 0.62 mg/L during pumping compared to pre-crowding levels (Table 5). Larger catch sizes generally resulted in a greater reduction in DO_2_ (Fig. 5, df = 1, *F* = 26.37, *p* < 0.001, R^2^ = 0.70, Supplementary Table S8). One exception to this was catch “L02” (refer to Table 5), which experienced the third largest drop in DO_2_ (− 1.97 mL/L) but with a biomass of only 81.6 tonnes (Fig. 5).

**Figure 6:**
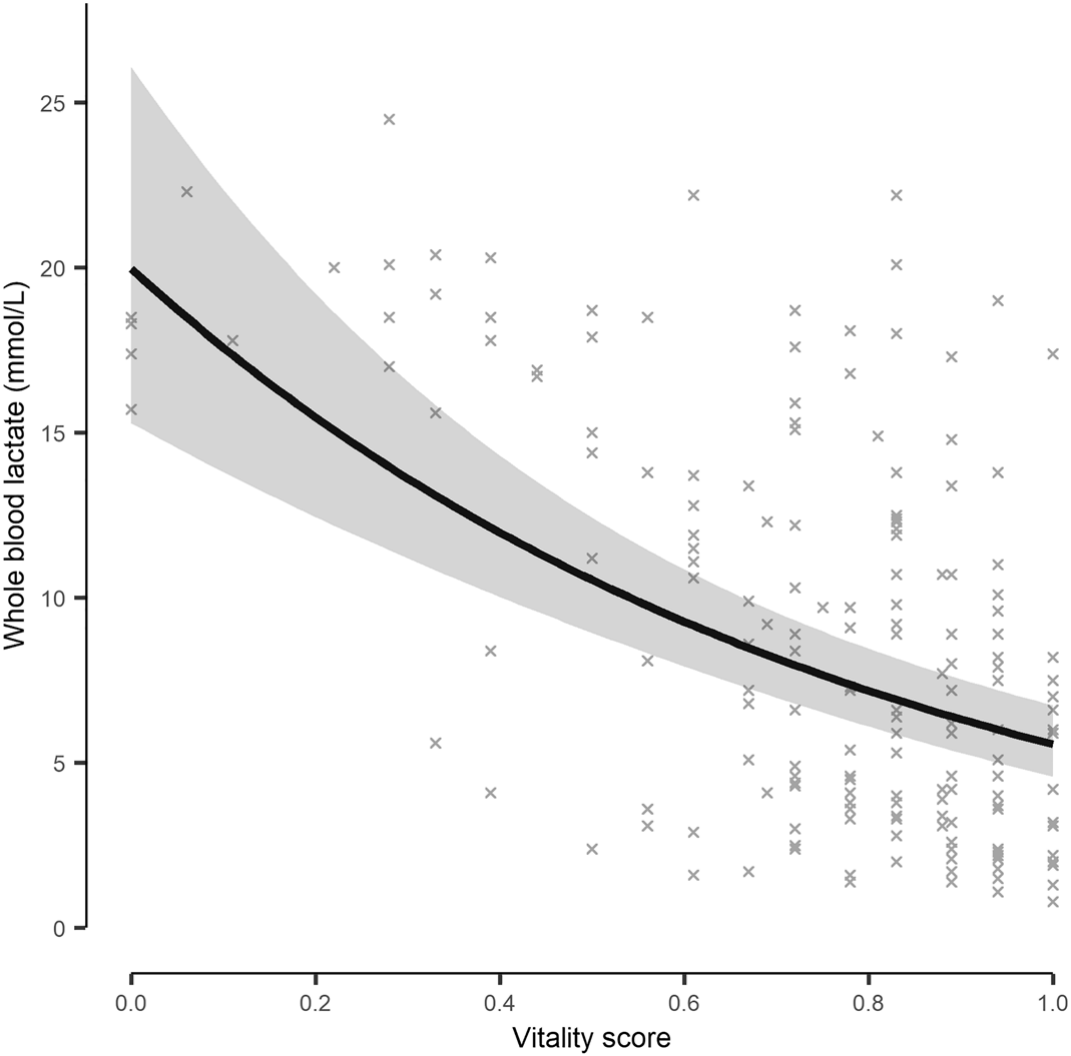
The model derived relationship between vitality scores and blood lactate for Atlantic mackerel (*Scomber scombrus*) during crowding events onboard a commercial purse seine vessel. Fish were collected throughout crowding (after being pumped onboard), assessed for behavioural vitality and then had blood collected from the caudal vasculature. Lactate was quantified using a Lactate Pro 2 point of care device. The shaded area indicates the model derived 95% confidence interval, with the underlying data indicated as points.

Of the 205 fish assessed for vitality, ~ 84% also had blood collected. Blood lactate concentrations were generally low (< 10 mmol/L) at the beginning of pumping but increased towards the end (Supplementary Figure S5). The range of values observed was between 0.8 and 24.5 mmol/L. The VS were significantly related to lactate concentrations (df = 1, LRT = 50.23, *p* < 0.001, Supplementary Table S9). Mackerel with lower vitality scores tended to have higher levels of blood lactate than more vital fish, but with considerable variation between individuals (Fig. 6).

For the other candidate drivers of VS, the model ranking procedure indicated equal support (i.e. ΔAICc < 2) for the top three competing models in each group (refer to Table 6 for ranking of the model group considering the DO_2_ 1 min average, and Supplementary Tables S16 and S17 for the 3 and 5 min DO_2_ groups). Together, these top models contained main effects of all the candidate variables (Table 6).

**Table 6:** Ranking of candidate generalized linear mixed models (beta error structures with logit link) to explain the vitality of Atlantic mackerel (*Scomber scombrus*) during pumping related crowding during purse seine capture.

Rank	Fixed effect variables	Log likelihood	ΔAICc	Weight
1	Exposure time + Pumping rate	83.961	0.000	0.270
2	Exposure time + Pumping rate + Pump depth	84.978	0.140	0.252
3	Exposure time + Pumping rate + DO_2_ (1 min)	84.388	1.320	0.139
4	Exposure time + Pumping rate + Exposure time × Pumping rate	83.962	2.170	0.091
5	Exposure time + Pumping rate + Pump depth + DO_2_ (1 min)	85.029	2.230	0.088
6	Exposure time + Pumping rate + Pump depth + Exposure time × Pumping rate	84.980	2.330	0.084
7	Exposure time + Pumping rate + DO_2_ (1 min) + Exposure time × Pumping rate	84.392	3.510	0.047
8	Exposure time + Pumping rate + Pump depth + DO_2_ (1 min) + Exposure time × Pumping rate	85.033	4.440	0.029

The “DO_2_ (1 min)” term indicates a moving average of dissolved oxygen conditions in the net 1 min prior to vitality sampling. AICc refers to Akaike Information Criteria (corrected for small sample size) and “Weight” to normalised Akaike weights.

**Figure 7:**
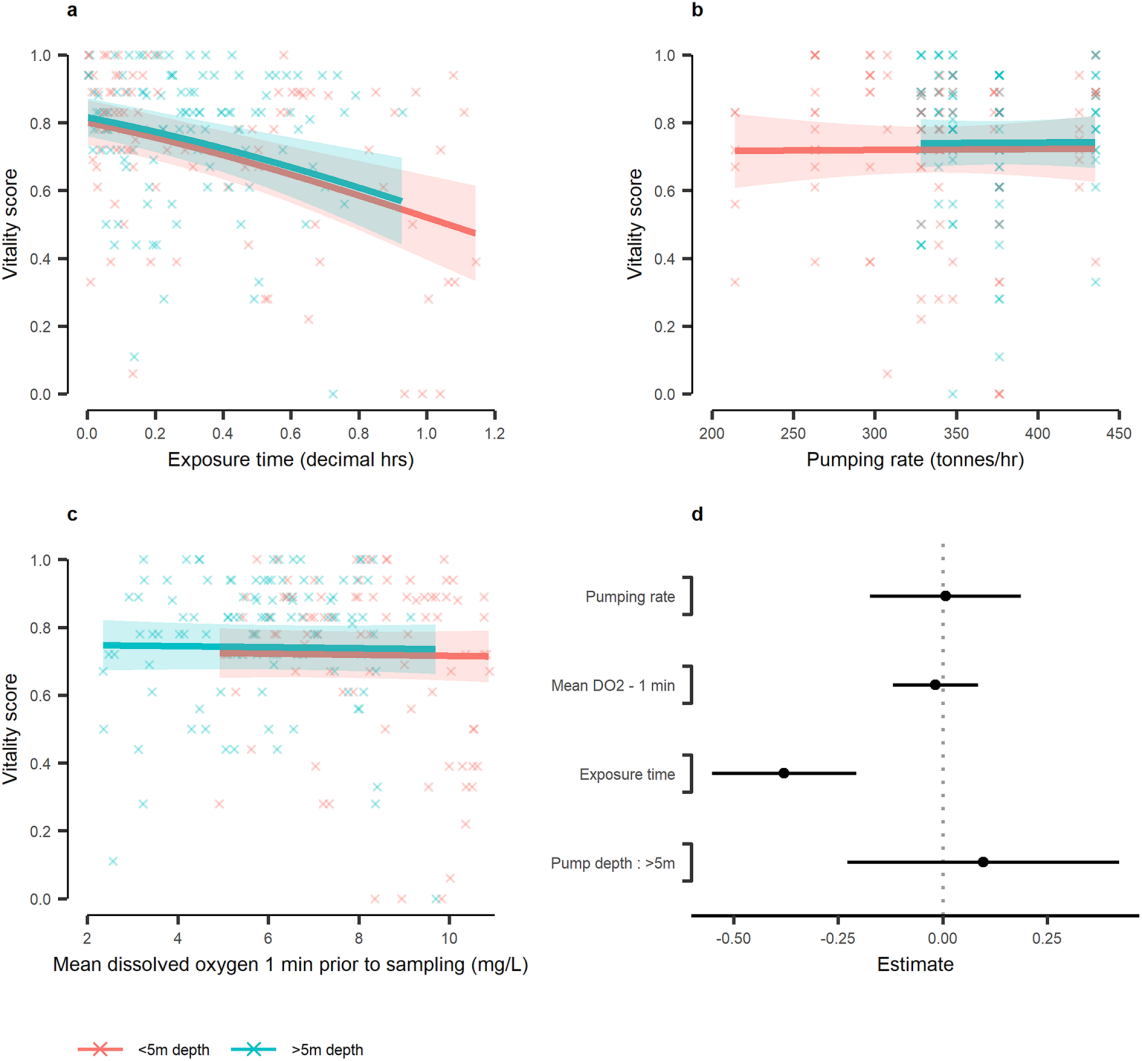
The model derived relationship between vitality scores and various drivers during crowding-related pumping of Atlantic mackerel (*Scomber scombrus*) during commercial purse seine capture. The model fits were constructed from averaging of three top competing models selected by Akaike information criterion. Fish were collected throughout crowding (after being pumped onboard) and assessed for behavioural vitality. (**a**) The relationship with crowding exposure time, for the mean pumping rate (351 tonnes/h) and mean dissolved oxygen (DO_2_) in the net during the previous 1 min prior to vitality sampling (6.95 mg/L). (**b**) The relationship with pumping rate, for the mean exposure time (0.33 h) and mean DO_2_. (**c**) The relationship with DO_2_ in the net during the previous 1 min prior to vitality sampling, for the mean exposure time and mean pumping rate. The shaded area indicates the model derived 95% confidence interval, with the underlying data indicated as crosses. All relationships are factorised according to pump depth (either < 5 m depth or > 5 m depth). (**d**) Model coefficients (black points) for the averaged model, with 95% confidence intervals as whiskers. The dotted grey line indicates zero effect. All continuous model covariates were scaled prior to fitting.

The outcome of model averaging of the top three models for 1 min DO_2_ group is illustrated in Fig. 7. The 95% confidence intervals around the coefficient estimates indicated that only exposure time had a significant effect on vitality (Fig. 7d). The model (Supplementary Table S11) predicted that the longer a mackerel was exposed to crowding, the lower its vitality; for each hour of exposure a ~ 35% reduction in vitality could be expected (Fig. 7a). Mackerel pumped from less than 5 m depth tended to have lower vitality (by ~ 2%, Fig. 7) but the effect was not significant (Fig. 7d). Otherwise, pumping rate and DO_2_ had negligible (Fig. 7b,c) and non-significant (Fig. 7d) impacts on VS. These findings matched those for the 3 and 5 min DO_2_ model groups (Supplementary Figs S6, S7; Supplementary Tables S16, S17).

## Discussion

In this study, we used vitality assessment to determine how pumping-related crowding influences the welfare of mackerel during commercial purse seine fishing operations. The results of cage trials demonstrated that vitality was an acceptable measure of welfare for two reasons. Firstly, the magnitude of the vitality response was determined by the intensity (i.e. the density) and duration (i.e. the exposure time) of the crowding stress that individual fish received, with no appreciable response in the non-crowded controls. The reduction in vitality also occurred rapidly, during the stressor exposure itself. These characteristics match the definition of an informative stress indicator^9,10^. Secondly, the cage trial data showed that individual fish welfare scores (generated from multivariate analysis on a suite of established stress metrics) could be predicted using vitality. Although there was considerable variation between individual fish, lower vitality scores (VS) were generally indicative of a lower welfare state. There is a growing body of literature in which the vitality of fish has been assessed in the context of fisheries capture and the associated mortality of discards^28^. However, our study represents the first attempt at correlating multivariate welfare scores to vitality.

Functional welfare is the multifaceted expression of a variety of stress responses^1^. We attempted to encompass this by integrating a range of neuro-endocrine, physiological and physical metrics into a single welfare score using multivariate analysis. This approach has been used previously in aquaculture settings^21,22^. The PCA loadings in the cage trials were consistent with previous work on mackerel stress responses to crowding^41,44^ and correlated to whether fish were crowded or were recovering. Lower welfare scores were also evident for trials in which mortality subsequently occurred, demonstrating a link between the welfare scores we generated and important fitness outcomes. Together, these findings demonstrate the PCA scores were an acceptable means of quantifying welfare in mackerel.

Although we were unable to measure the vitality of uncrowded fish prior to capture during our field observations due to logistical constraints, it is fair to assume that wild, uncrowded mackerel have high to full vitality. This was evident from the high vitality we observed for control fish in the cage trials and fish sampled at the start of the pumping process onboard the vessel. The fact that lower vitality fish tended to have higher lactate levels suggests a similar physiological response between our at-sea observations and the cage trial simulations^41^. Based on the demonstrated relationship between vitality and welfare, the changes in vitality we observed at sea therefore indicate that the pumping related crowding can negatively impact the welfare of mackerel. It is, however, not possible to objectively infer exactly how badly welfare was impacted for individual catches or fish. To do so would require the welfare scores we generated to be correlated to important fitness outcomes such as long term sub-lethal consequences or individual mortality^53^. Given that the large size of the sea cages and the numbers of fish we used, it was not possible to monitor such outcomes at the individual level. Future work in this area should therefore attempt to correlate individual welfare and VS to survival outcomes using experimental setups that allow individual fish to be monitored and that employ a wider range of applied densities and/or durations.

Our modelling of potential drivers of welfare in fisheries catches indicated that mackerel vitality was determined by their exposure time to high density crowding in the net during pumping, but not on crowding density (as measured by our proxy of pumping rates). This is inconsistent with the findings of our cage trial and the theoretical understanding of stress responses, where both duration and intensity of a stressor should determine the response^55^. It could be that our pumping rate proxy did not accurately characterise the density experienced by fish in the net, or that that the range of crowding densities in the catches was not large enough to induce a measurable VS response. Given the relatively small variance in pumping rates we observed, and the fact that fishers attempt to maintain an optimal pumping density by adjusting net volume, the later explanation seems the more plausible.

The vitality metrics we monitored (Table 2) should have been responsive to changes in dissolved oxygen concentration (DO_2_)^67–69^, but there was no evidence that this variable significantly influenced vitality in either the cage trials or the pumped catches. The hypoxic threshold of mackerel is currently unknown, but the species is oxyphilic^70^. Although less influential than crowding, reductions in oxygen do cause increases in behavioural activity in mackerel^42^. It is therefore somewhat surprising that oxygen did not play a more important role in determining welfare, especially considering that some catches experienced concentrations of < 4 mg/L for several minutes (Supplementary Fig. S5). It could be that the hypoxic tolerance of mackerel is higher than their metabolic rates would indicate. Indeed, cage trials have shown that mackerel can tolerate short-term (< 1 h) exposure to reduced DO_2_ concentrations (~ 4.5 mg/L) with no appreciable mortality^53^. Our method of monitoring of oxygen levels could also have not accurately reflected the conditions that individual fish experienced. Considering the uncontrolled and highly dynamic conditions inside the crowded cages and nets at sea, this would seem plausible. We propose that future work attempts to define hypoxic thresholds in Atlantic mackerel to better understand the potential of this stressor to impact upon welfare during wild capture.

Other than exposure time, catch size was also found to play a role in determining mackerel vitality in pumped catches. DO_2_, crowding exposure and pumping rates were, however, all highly correlated with this variable. The lack of any significant effect of DO_2_ and pumping rate (our proxy for crowding density) during the modelling procedures suggests exposure time is the main underlying factor driving the catch size–VS relationship. Indeed, it is intuitive that larger catches take longer to pump onboard and result in longer exposure times. Purse seine vessels typically use acoustic methods to determine biomass before setting the net. Although this method comes with a some degree of error, catch size control is relatively achievable if best practise is followed^71^. Therefore, the relationship between catch size and vitality demonstrated here may provide an operational means by which catch welfare can be controlled.

Consistent with our POC device measurements of blood lactate taken at sea, the physiological response to crowding in mackerel is characterized by large increases in lactate concentrations, the magnitude of which is dependent on crowding density and exposure time^41^. In terms of behavioural responses, crowding causes increased activity even at relatively low density levels^42^. Anecdotal reports indicate that large mackerel catches are routinely seen to “boil” during crowding, with uncoordinated and high intensity escape responses being exhibited^51^. Together, these observations suggest that pumping related crowding can result in high-intensity anaerobic locomotory activity in mackerel to such an extent that welfare is negatively impacted^72^. High swimming activity in response to stressful situations has been observed in mackerel in other scenarios^73^ and likely represents an adaptive response that would lessen stressor exposure duration in a naturally occurring scenario. Such a response is, however, rendered maladaptive in a capture situation where escape through the small meshes of the purse seine is not possible. At extreme densities, close contact with other fish may hinder efforts to ram ventilate or even prevent fish from performing buccal pumping. This would further contribute to physiological disturbance via respiratory acidosis. Physiological changes induced by crowding, including elevated lactate levels, are associated with negative impacts on mackerel flesh quality^44,74^, and flesh quality may in turn determine the price of catches to some extent^75^. Establishing a link between welfare scores, vitality and flesh quality should therefore be a key component of future work, so that fishers are incentivised to consider catch welfare as a means of maximizing profitability^4^.

In larger catches, pump depth generally decreased sharply towards the end of pumping (Supplementary Fig. S5), as net volume was reduced to empty any remaining catch. Although not significant, our models indicated that fish collected from < 5 m tended to have lower vitality. Mackerel are negatively buoyant and sink if they are exhausted and unable to swim^73^. We therefore suggest that the reduced vitality of fish collected from when the pump depth was < 5 m in larger catches resulted from a combination of longer exposure times and an increased likelihood of encountering exhausted individuals. However, such effects would be conditional on catch size. In smaller catches, exposure times were short with pumping depths < 5 m throughout. This conflation likely contributed to the lack of significance of pumping depth in our models.

There was considerable variance in the vitality, welfare scores and physiological responses of individual fish for both the cages and field observations. Our modelling results indicate that at least some of this variability could be attributed to differences in crowding density and exposure time at the individual level. For instance, camera observations from inside purse seine nets indicate crowding levels are not uniform for all fish as localized areas of high density can occur^76^. The response to stress is also highly specific to the affected individual and depends upon a variety of intrinsic and extrinsic factors such as sex, feeding status, season, motivation, conspecific activities, prior experiences and coping styles^11^. The diversity of factors potentially affecting the response of mackerel to stress highlights that consistently managing welfare impacts during wild capture is likely to be a difficult task. It is also important to note that the mean catch size we observed during our field observations was relatively modest^38^ and was undertaken on only one vessel. It would therefore be informative for future work to observe vitality/welfare in a larger range of catch sizes and from a wider variety of vessels, to help determine how representative our observations are of the wider fishery.

In conclusion, the results indicate that mackerel welfare can be negatively impacted by pumping related crowding during purse seine capture. It is probable that high-intensity anaerobic locomotory activity is responsible for the welfare reduction. Welfare conscious fishing practices can be defined as those that minimize the allostatic load on fish until they are released or slaughtered^4^. Consequently, if the negative welfare impacts of pumping are to be mitigated, the stressor (crowding) or its response (intense activity followed by physiological disturbance) must be minimized. Theoretically, the response could be avoided by humanely stunning and/or slaughtering the fish in the net before crowding becomes too intense. It is difficult, however, to envisage how this could be practically accomplished given the large numbers of fish involved and the lack of suitable technological solutions. More feasible would be to control the crowding stressor itself. Our results demonstrate better welfare for shorter crowding exposure times. Therefore, pumping catches onboard as rapidly as possible should, theoretically, improve catch welfare. Unfortunately, there is limited scope for this because fishers are already incentivised to pump at high rates to minimise vessel operating costs. Additional physical forces generated by high-speed pumping could also impact welfare. Without the development of new benign and low-density pumping technology, alternative ways of avoiding long exposure times should therefore be sought. Our results indicate that larger catches experience longer exposure times. We therefore suggest that fishers target smaller school sizes to promote animal welfare during wild capture purse seine fishing.

## Supplementary Information

Supplementary Information.

## Data Availability

The data underlying this article will be shared upon reasonable request to the corresponding author.
