# Supplementary material for: Vitality as a measure of animal welfare during purse seine pumping related crowding of Atlantic mackerel (Scomber scrombrus)
Source: Sci Rep. 2022 Dec 19;12:21949. doi: 10.1038/s41598-022-26373-x (PMC9763418; doi:10.1038/s41598-022-26373-x)
Supplement: Supplementary file 1 — Supplementary Information. [file 41598_2022_26373_MOESM1_ESM.pdf]

# Supplementary Information

**Vitality as a measure of animal welfare during purse seine pumping-related crowding of Atlantic mackerel (*Scomber scrombrus*).**

**Neil Anders, Sigurd Hannaas, Jostein Saltskår, Erik Schuster, Maria Tenningen, Bjørn Totland, Aud Vold, Jan Tore Øvredal & Mike Breen**

---

This document contains the following:

- Supplementary Table S1: Coefficient table - pp 2
- Supplementary Table S2: Coefficient table - pp 3
- Supplementary Table S3: Coefficient table - pp 4
- Supplementary Table S4: Coefficient table - pp 5
- Supplementary Table S5: Coefficient table - pp 6
- Supplementary Table S6: Coefficient table - pp 7
- Supplementary Table S7: Coefficient table - pp 8
- Supplementary Table S8: Coefficient table - pp 9
- Supplementary Table S9: Coefficient table - pp 10
- Supplementary Table S10: Coefficient table - pp 11
- Supplementary Table S11: Coefficient table - pp 12
- Supplementary Table S12: Coefficient table - pp 13
- Supplementary Table S13: Coefficient table - pp 14

- Supplementary Table S14: Model ranking table - pp 15
- Supplementary Table S15: Model ranking table - pp 16
- Supplementary Table S16: Model ranking table - pp 17
- Supplementary Table S17: Model ranking table - pp 18

- Supplementary Figure S1: pp 19
- Supplementary Figure S2: pp 20
- Supplementary Figure S3: pp 21
- Supplementary Figure S4: pp 22
- Supplementary Figure S5: pp 23 – pp 25
- Supplementary Figure S6: pp 26
- Supplementary Figure S7: pp 27

**Table S1**: Coefficient estimates from a linear model to describe the relationship between vitality scores generated from two different procedures of Atlantic mackerel (*Scomber scrombrus*) during cage trials; either “handling-only” or “while handling and free swimming”.

| | Value | Std. Error | t-value | p-value |
|---|---|---|---|---|
| Intercept | -0.025 | 0.026 | -0.949 | 0.344 |
| “Handling only” vitality scores | 0.998 | 0.030 | 33.738 | <0.001 |

**Table S2**: Coefficient estimates from a generalised least square (GLS) model to describe the relationship between Principal Component 1 from a Hill and Smith principal component analysis and the status of Atlantic mackerel (*Scomber scrombrus*) in cage crowding trials. "Recovered" status refers to fish sampled at the end of survival monitoring.

| | **Value** | **Std. Error** | **t-value** | **p-value** |
|---|---|---|---|---|
| During crowding (intercept) | 2.107 | 0.361 | 5.838 | <0.001 |
| 2hrs after crowding | -1.768 | 0.507 | -3.486 | 0.001 |
| 24hrs after crowding | -2.938 | 0.401 | -7.331 | <0.001 |
| Recovered | -3.673 | 0.406 | -9.048 | <0.001 |
| Not crowded | -2.862 | 0.368 | -7.775 | <0.001 |
| Moribund | -1.219 | 0.617 | -1.976 | 0.050 |

**Table S3**: Coefficient estimates from a generalised least square (GLS) model to describe the relationship between Principal Component 1 from a Hill and Smith principal component analysis and different Atlantic mackerel (*Scomber scrombrus*) cage crowding trials.

| | Value | Std. Error | t-value | p-value |
|---|---|---|---|---|
| Control 1 (intercept) | -0.438 | 0.134 | -3.274 | 0.002 |
| Control 2 | -0.031 | 0.228 | -0.135 | 0.893 |
| Control 3 | -0.410 | 0.253 | -1.619 | 0.111 |
| Low | -0.421 | 0.238 | -1.769 | 0.083 |
| Moderate | 0.720 | 0.398 | 1.808 | 0.076 |
| High | 1.683 | 0.463 | 3.639 | 0.001 |

**Table S4**: Coefficient estimates from a generalised least square (GLS) model to describe the relationship between Principal Component 2 from a Hill and Smith principal component analysis and the status of Atlantic mackerel (*Scomber scrombrus*) in cage crowding trials. "Recovered" status refers to fish sampled at the end of survival monitoring.

| | Value | Std. Error | t-value | p-value |
|---|---|---|---|---|
| During crowding (intercept) | 0.186 | 0.247 | 0.751 | 0.454 |
| 2hrs after crowding | 0.760 | 0.328 | 2.315 | 0.022 |
| 24hrs after crowding | 0.105 | 0.339 | 0.310 | 0.757 |
| Recovered | -0.030 | 0.355 | -0.084 | 0.933 |
| Not crowded | -0.381 | 0.288 | -1.324 | 0.187 |
| Moribund | -1.286 | 0.553 | -2.324 | 0.021 |

**Table S5**: Coefficient estimates from a generalised least square (GLS) model to describe the relationship between Principal Component 3 from a Hill and Smith principal component analysis and the status of Atlantic mackerel (*Scomber scrombrus*) in cage crowding trials. "Recovered" status refers to fish sampled at the end of survival monitoring.

| | Value | Std. Error | t-value | p-value |
|---|---|---|---|---|
| During crowding (intercept) | -0.321 | 0.135 | -2.386 | 0.018 |
| 2hrs after crowding | 0.487 | 0.314 | 1.551 | 0.123 |
| 24hrs after crowding | 0.016 | 0.287 | 0.057 | 0.954 |
| Recovered | 1.496 | 0.435 | 3.441 | 0.001 |
| Not crowded | -0.079 | 0.154 | -0.516 | 0.607 |
| Moribund | 5.071 | 0.280 | 18.090 | <0.001 |

**Table S6**: Coefficient estimates from a generalised linear model (beta error structure) to describe the relationship between Atlantic mackerel (*Scomber scrombrus*) vitality scores and crowding exposure time for various cage crowding trials. Estimates are presented on the logit scale.

| | **Estimate** | **Std. Error** | **z value** | **p-value** |
|---|---|---|---|---|
| Trial : Control 2 (intercept) | 4.536 | 0.390 | 11.642 | < 0.001 |
| Trial : Control 3 | -1.428 | 0.556 | -2.570 | 0.010 |
| Trial : High | -2.840 | 0.568 | -5.001 | <0.001 |
| Trial : High & Prolonged 1 | -3.562 | 0.434 | -8.200 | <0.001 |
| Trial : High & Prolonged 2 | -2.917 | 0.413 | -7.064 | <0.001 |
| Trial : Low | -1.509 | 0.632 | -2.387 | 0.017 |
| Trial : Moderate | -1.764 | 0.632 | -2.790 | 0.005 |
| Exposure time | -1.256 | 0.406 | -3.093 | 0.002 |

**Table S7**: Coefficient estimates from a linear mixed model to describe the relationship between Principal Component 1 from a Hill and Smith principal component analysis and the vitality of Atlantic mackerel (*Scomber scrombrus*) in various crowding trials conducted in aquaculture cages.

| **Random effects** | | | **Fixed effects** | | | | |
|---|---|---|---|---|---|---|---|
| | **Variance** | **Std. Dev** | | **Estimate** | **Std. Error** | **z value** | **p-value** |
| Vitality score (slope) | 1.829 | 1.352 | Intercept | -4.093 | 0.631 | -6.489 | <0.001 |
| | | | Vitality score | 4.277 | 0.839 | 5.098 | <0.001 |

**Table S8:** Coefficient estimates from a linear model to describe the relationship between catch size and reductions in dissolved oxygen inside the catch during Atlantic mackerel (*Scomber scrombrus*) purse seine pumping events.

| | **Estimate** | **Std. Error** | **t value** | **p-value** |
|---|---|---|---|---|
| (Intercept) | 0.349 | 0.264 | 1.323 | 0.215 |
| Catch size | 0.006 | 0.001 | 5.135 | <0.001 |

**Table S9**: Coefficient estimates from a generalised linear mixed model (gamma error structure with log link) to describe the relationship between blood lactate levels (as measured by a point-of-care device) during pumping of catches onboard a commercial fishing vessel and the vitality of Atlantic mackerel (*Scomber scrombrus*). Estimates are presented on the log scale.

| **Random effects** | | | **Fixed effects** | | | | |
|---|---|---|---|---|---|---|---|
| | **Variance** | **Std. Dev** | | **Estimate** | **Std. Error** | **z value** | **p-value** |
| Catch : Trip (intercept) | 0.0285 | 0.169 | Intercept | 2.994 | 0.136 | 21.997 | <0.001 |
| Trip (intercept) | <0.001 | <0.001 | Vitality score | -1.277 | 0.167 | -7.619 | <0.001 |

**Table S10**: Coefficient estimates from a generalised linear mixed model (beta error structure with logit link) to describe the relationship between catch size and the vitality of Atlantic mackerel (*Scomber scrombrus*) during pumping related crowding. Estimates are presented on the logit scale.

| Random effects | | | Fixed effects | | | | |
|---|---|---|---|---|---|---|---|
| | **Variance** | **Std. Dev** | | **Estimate** | **Std. Error** | **z value** | **p-value** |
| Catch : Trip (intercept) | 0.0484 | 0.220 | Intercept | 0.933 | 0.112 | 8.451 | <0.001 |
| Trip (intercept) | <0.001 | <0.001 | Catch size | -0.190 | 0.105 | -1.802 | 0.716 |

**Table S11**: Coefficient estimates from averaging of three top competing generalised linear mixed models (beta error structure with logit link) to describe the relationship between various potential drives and the vitality of Atlantic mackerel (*Scomber scrombrus*) during pumping related crowding. Estimates are presented on the logit scale, and covariates were scaled according to their mean and SD before fitting. "$DO_2$ (1 min)" indicates the mean dissolved oxygen concentration (mg/L) experienced by fish in the previous 1 minute prior to vitality sampling. AICw refers to normalised Akaike weights, here summed across the three averaged models. "Proportion of containing models" refer to the proportion of the three averaged models that contained the given coefficient.

| | **Estimate** | **Std. Error** | **Adjusted SE** | **z value** | **p-value** | **Sum of AICw** | **Proportion of containing models** |
|---|---|---|---|---|---|---|---|
| Pump depth : < 5m (intercept) | 0.947 | 0.174 | 0.175 | 5.414 | <0.001 | NA | 1.00 |
| Exposure time | -0.379 | 0.087 | 0.088 | 4.308 | <0.001 | 1.00 | 1.00 |
| Pumping rate | 0.007 | 0.091 | 0.092 | 0.077 | 0.938 | 1.00 | 1.00 |
| Pump depth : > 5m | 0.097 | 0.165 | 0.166 | 0.585 | 0.559 | 0.38 | 0.33 |
| $DO_2$ (1 min) | -0.017 | 0.052 | 0.052 | 0.327 | 0.744 | 0.15 | 0.33 |

**Table S12**: Coefficient estimates from averaging of three top competing generalised linear mixed models (beta error structure with logit link) to describe the relationship between various potential drives and the vitality of Atlantic mackerel (*Scomber scrombrus*) during pumping related crowding. Estimates are presented on the logit scale, and covariates were scaled according to their mean and SD before fitting. “$DO_2$ (3 mins)” indicates the mean dissolved oxygen concentration (mg/L) experienced by fish in the previous 3 minutes prior to vitality sampling. AICw refers to normalised Akaike weights, here summed across the three averaged models. “Proportion of containing models” refer to the proportion of the three averaged models that contained the given coefficient.

| | **Estimate** | **Std. Error** | **Adjusted SE** | **z value** | **p-value** | **Sum of AICw** | **Proportion of containing models** |
|---|---|---|---|---|---|---|---|
| Pump depth : < 5m (intercept) | 0.944 | 0.174 | 0.175 | 5.385 | 0.000 | NA | 1.00 |
| Exposure time | -0.382 | 0.087 | 0.088 | 4.358 | 0.000 | 1.00 | 1.00 |
| Pumping rate | 0.010 | 0.092 | 0.092 | 0.107 | 0.914 | 1.00 | 1.00 |
| Pump depth : > 5m | 0.102 | 0.168 | 0.169 | 0.607 | 0.544 | 0.40 | 0.33 |
| $DO_2$ (3 mins) | -0.008 | 0.038 | 0.039 | 0.196 | 0.844 | 0.17 | 0.33 |

**Table S13**: Coefficient estimates from averaging of three top competing generalised linear mixed models (beta error structure with logit link) to describe the relationship between various potential drives and the vitality of Atlantic mackerel (*Scomber scrombrus*) during pumping related crowding. Estimates are presented on the logit scale, and covariates were scaled according to their mean and SD before fitting. "$DO_2$ (5 mins)" indicates the mean dissolved oxygen concentration (mg/L) experienced by fish in the previous 5 minutes prior to vitality sampling. AICw refers to normalised Akaike weights, here summed across the three averaged models. "Proportion of containing models" refer to the proportion of the three averaged models that contained the given coefficient.

| | **Estimate** | **Std. Error** | **Adjusted SE** | **z value** | **p-value** | **Sum of AICw** | **Proportion of containing models** |
|---|---|---|---|---|---|---|---|
| Pump depth : < 5m (intercept) | 0.943 | 0.174 | 0.175 | 5.375 | 0.000 | NA | 1.00 |
| Exposure time | -0.383 | 0.087 | 0.088 | 4.374 | 0.000 | 1.00 | 1.00 |
| Pumping rate | 0.011 | 0.092 | 0.092 | 0.124 | 0.901 | 1.00 | 1.00 |
| Pump depth : > 5m | 0.105 | 0.169 | 0.170 | 0.615 | 0.538 | 0.41 | 0.33 |
| $DO_2$ (5 mins) | -0.002 | 0.032 | 0.032 | 0.057 | 0.955 | 0.15 | 0.33 |

**Table S14**: Ranking of candidate generalized linear mixed models (beta error structures with logit link) to explain the vitality of Atlantic mackerel (*Scomber scombrus*) during sea cage crowding trials. The "$DO_2$ (3 min)" term indicates a moving average of dissolved oxygen conditions in the cage 3 mins prior to vitality sampling. AICc refers to Akaike Information Criteria (corrected for small sample size) and "Weight" to normalised Akaike weights.

| **Rank** | **Fixed effect variables** | **Log likelihood** | **ΔAICc** | **Weight** |
|---|---|---|---|---|
| 1 | Exposure time + Trial | 42.796 | 0.000 | 0.681 |
| 2 | Exposure time + Trial + $DO_2$ (3 min) | 42.945 | 2.300 | 0.216 |
| 3 | Exposure time + Trial + Exposure time × Trial | 46.192 | 4.170 | 0.085 |
| 4 | Exposure time + Trial + $DO_2$ (3 min) + Exposure time × Trial | 46.193 | 7.160 | 0.019 |

**Table S15**: Ranking of candidate generalized linear mixed models (beta error structures with logit link) to explain the vitality of Atlantic mackerel (*Scomber scombrus*) during sea cage crowding trials. The “DO2 (5 min)” term indicates a moving average of dissolved oxygen conditions in the cage 5 mins prior to vitality sampling. AICc refers to Akaike Information Criteria (corrected for small sample size) and “Weight” to normalised Akaike weights.

| **Rank** | **Fixed effect variables** | **Log likelihood** | **ΔAICc** | **Weight** |
|---|---|---|---|---|
| 1 | Exposure time + Trial | 42.796 | 0.000 | 0.684 |
| 2 | Exposure time + Trial + $DO_2$ (5 min) | 42.919 | 2.350 | 0.211 |
| 3 | Exposure time + Trial + Exposure time × Trial | 46.192 | 4.170 | 0.085 |
| 4 | Exposure time + Trial + $DO_2$ (5 min) + Exposure time × Trial | 46.195 | 7.160 | 0.019 |

**Table S16**: Ranking of candidate generalized linear mixed models (beta error structures with logit link) to explain the vitality of Atlantic mackerel (*Scomber scombrus*) during pumping related crowding during purse seine capture. The "$DO_2$ (3 min)" term indicates a moving average of dissolved oxygen conditions in the net 3 mins prior to vitality sampling. AICc refers to Akaike Information Criteria (corrected for small sample size) and "Weight" to normalised Akaike weights.

| **Rank** | **Fixed effect variables** | **Log likelihood** | **ΔAICc** | **Weight** |
|---|---|---|---|---|
| 1 | Exposure time + Pumping rate | 83.961 | 0.000 | 0.284 |
| 2 | Exposure time + Pumping rate + Pump depth | 84.978 | 0.140 | 0.265 |
| 3 | Exposure time + Pumping rate + $DO_2$ (3 min) | 84.105 | 1.880 | 0.111 |
| 4 | Exposure time + Pumping rate + Exposure time × Pumping rate | 83.962 | 2.170 | 0.096 |
| 5 | Exposure time + Pumping rate + Pump depth + $DO_2$ (3 min) | 85.002 | 2.290 | 0.090 |
| 6 | Exposure time + Pumping rate + Pump depth + Exposure time × Pumping rate | 84.98 | 2.330 | 0.088 |
| 7 | Exposure time + Pumping rate + $DO_2$ (3 min) + Exposure time × Pumping rate | 84.106 | 4.080 | 0.037 |
| 8 | Exposure time + Pumping rate + Pump depth + $DO_2$ (3 min) + Exposure time × Pumping rate | 85.003 | 4.500 | 0.030 |

**Table S17**: Ranking of candidate generalized linear mixed models (beta error structures with logit link) to explain the vitality of Atlantic mackerel (*Scomber scombrus*) during pumping related crowding during purse seine capture. The “$DO_2$ (5 min)” term indicates a moving average of dissolved oxygen conditions in the net 5 mins prior to vitality sampling. AICc refers to Akaike Information Criteria (corrected for small sample size) and “Weight” to normalised Akaike weights.

| Rank | Fixed effect variables | Log likelihood | ΔAICc | Weight |
|---|---|---|---|---|
| 1 | Exposure time + Pumping rate | 83.961 | 0 | 0.28 |
| 2 | Exposure time + Pumping rate + Pump depth | 84.978 | 0.14 | 0.261 |
| 3 | Exposure time + Pumping rate + Pump depth + $DO_2$ (5 min) | 85.23 | 1.83 | 0.112 |
| 4 | Exposure time + Pumping rate + $DO_2$ (5 min) | 83.972 | 2.15 | 0.096 |
| 5 | Exposure time + Pumping rate + Exposure time × Pumping rate | 83.962 | 2.17 | 0.095 |
| 6 | Exposure time + Pumping rate + Pump depth + Exposure time × Pumping rate | 84.98 | 2.33 | 0.087 |
| 7 | Exposure time + Pumping rate + Pump depth + $DO_2$ (5 min) + Exposure time × Pumping rate | 85.232 | 4.05 | 0.037 |
| 8 | Exposure time + Pumping rate + $DO_2$ (5 min) + Exposure time × Pumping rate | 83.972 | 4.35 | 0.032 |

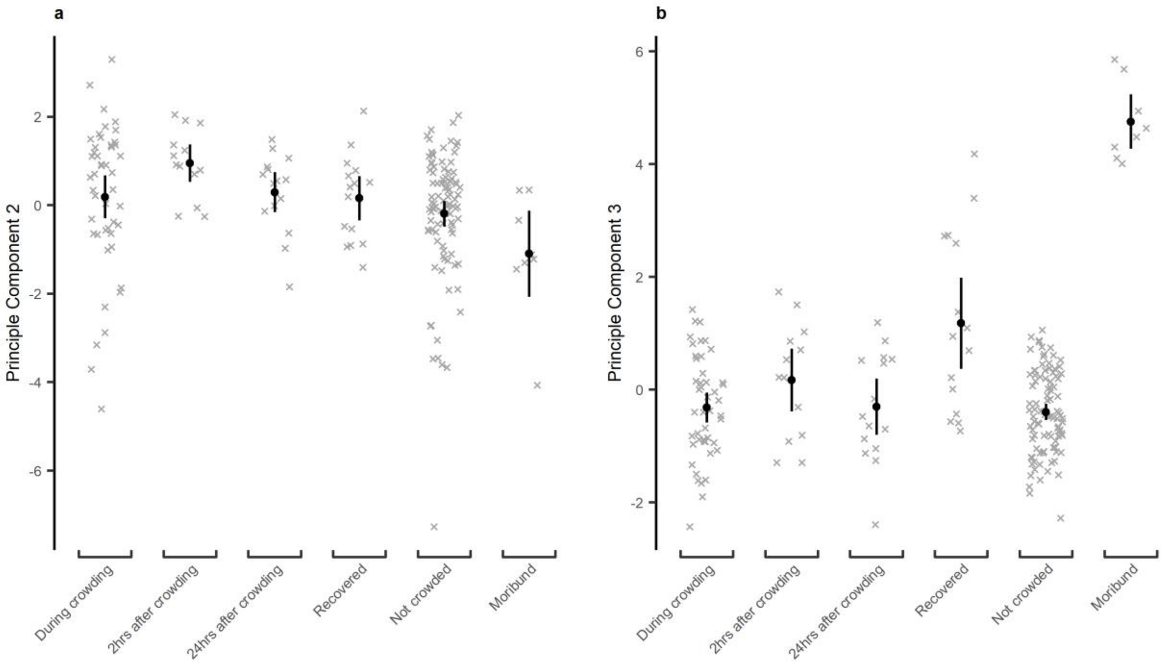


**Figure S1**: The relationship between treatment of Atlantic mackerel (*Scomber scrombrus*) sampled at various times during crowding trials and components from a Hill and Smith principal component analysis. **a**: the relationship with Principal Component 2. **b**: the relationship with Principal Component 3. Points indicate model derived mean values with 95% confidence intervals as whiskers. The underlying dataset is indicated as crosses.

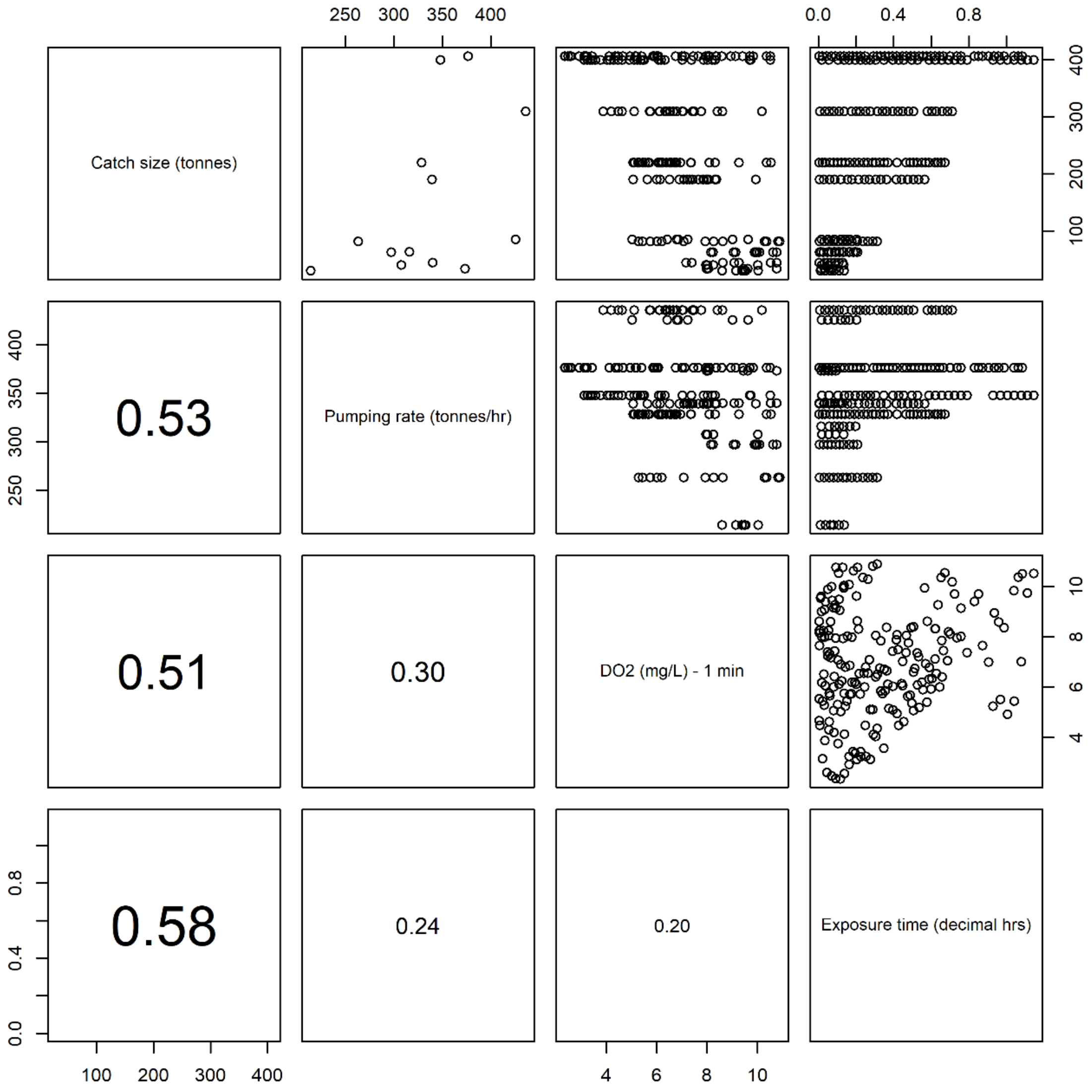


**Figure S2:** Collinearity between candidate continuous predictor variables to explain the vitality of Atlantic mackerel (*Scomber scrombrus*) during purse seine pumping-related crowding events. Upper panels show the raw data for each combination of variables; lower panels indicate the respective Pearson correlation coefficient, scaled in size to the value. "DO2 (mg/L) – 1 min" indicates the mean dissolved oxygen concentration (mg/L) experienced by fish in the previous minute prior to vitality sampling. The datasets are indicated with open circles.

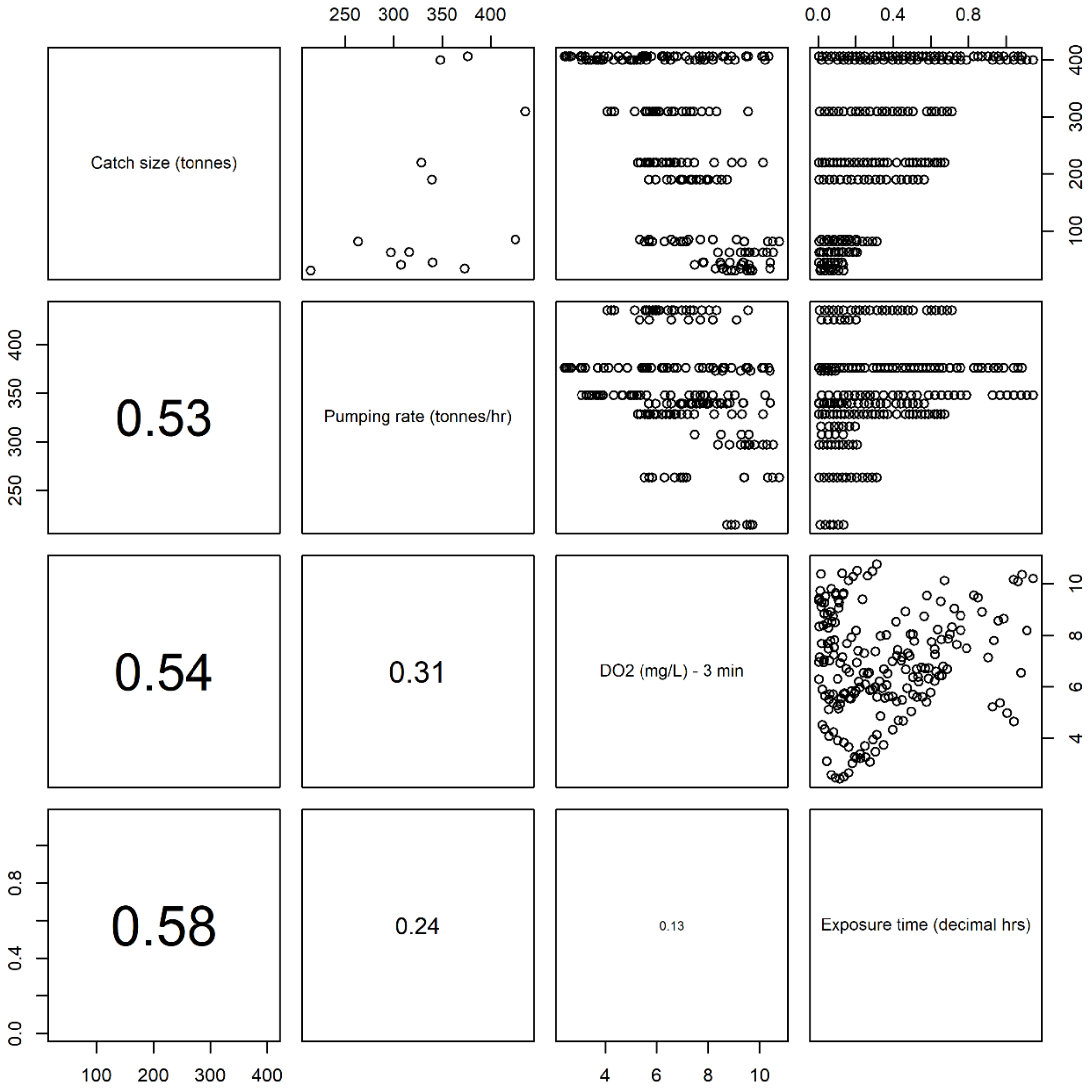


**Figure S3**: Collinearity between candidate continuous predictor variables to explain the vitality of Atlantic mackerel (*Scomber scrombrus*) during purse seine pumping-related crowding events. Upper panels show the raw data for each combination of variables; lower panels indicate the respective Pearson correlation coefficient, scaled in size to the value. "DO2 (mg/L) – 3 min" indicates the mean dissolved oxygen concentration (mg/L) experienced by fish in the previous 3 minutes prior to vitality sampling. The datasets are indicated with open circles.

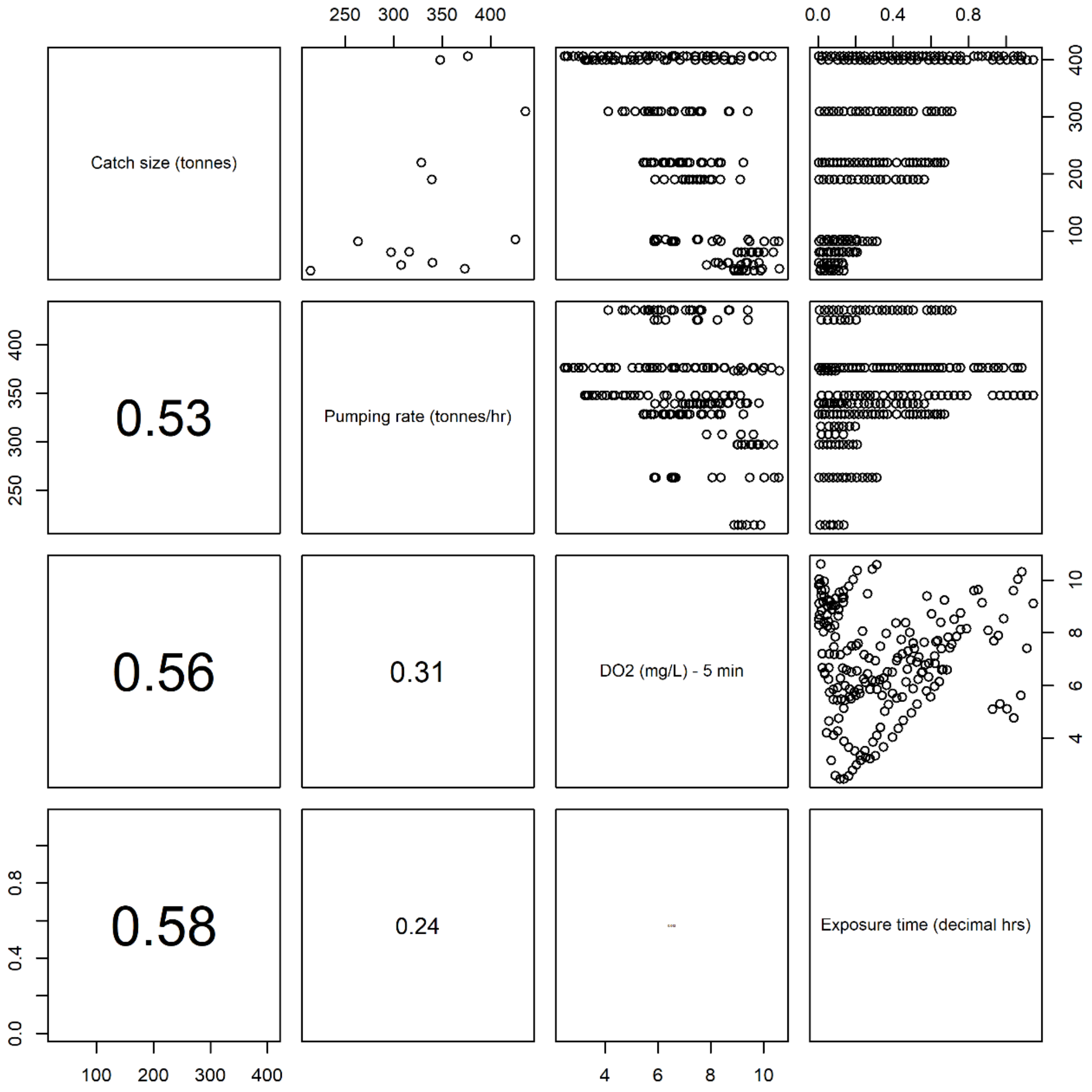


**Figure S4**: Collinearity between candidate continuous predictor variables to explain the vitality of Atlantic mackerel (*Scomber scrombrus*) during purse seine pumping-related crowding events. Upper panels show the raw data for each combination of variables; lower panels indicate the respective Pearson correlation coefficient, scaled in size to the value. “DO2 (mg/L) – 5 min” indicates the mean dissolved oxygen concentration (mg/L) experienced by fish in the previous 5 minutes prior to vitality sampling. The datasets are indicated with open circles.

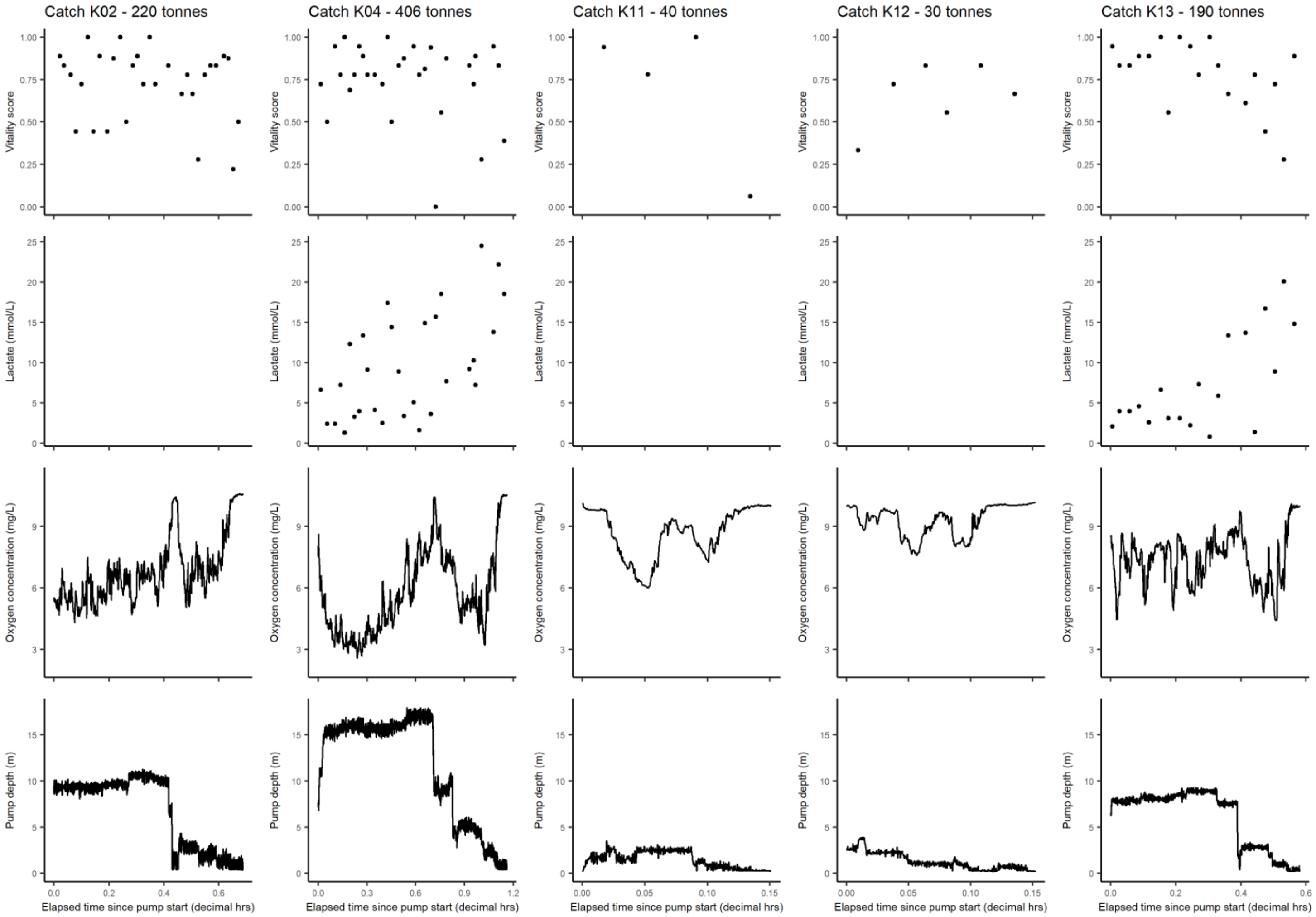


**Figure S5a**: Temporal change in pump depth and dissolved oxygen concentration (lower two panels) inside commercial purse seine catches of Atlantic mackerel (*Scomber scombrus*) during Trip 1 (refer to Table 5 in main article for further detail). Blood lactate and vitality scores from individual fish sampled throughout the pumping related crowding is also displayed (top two panels).

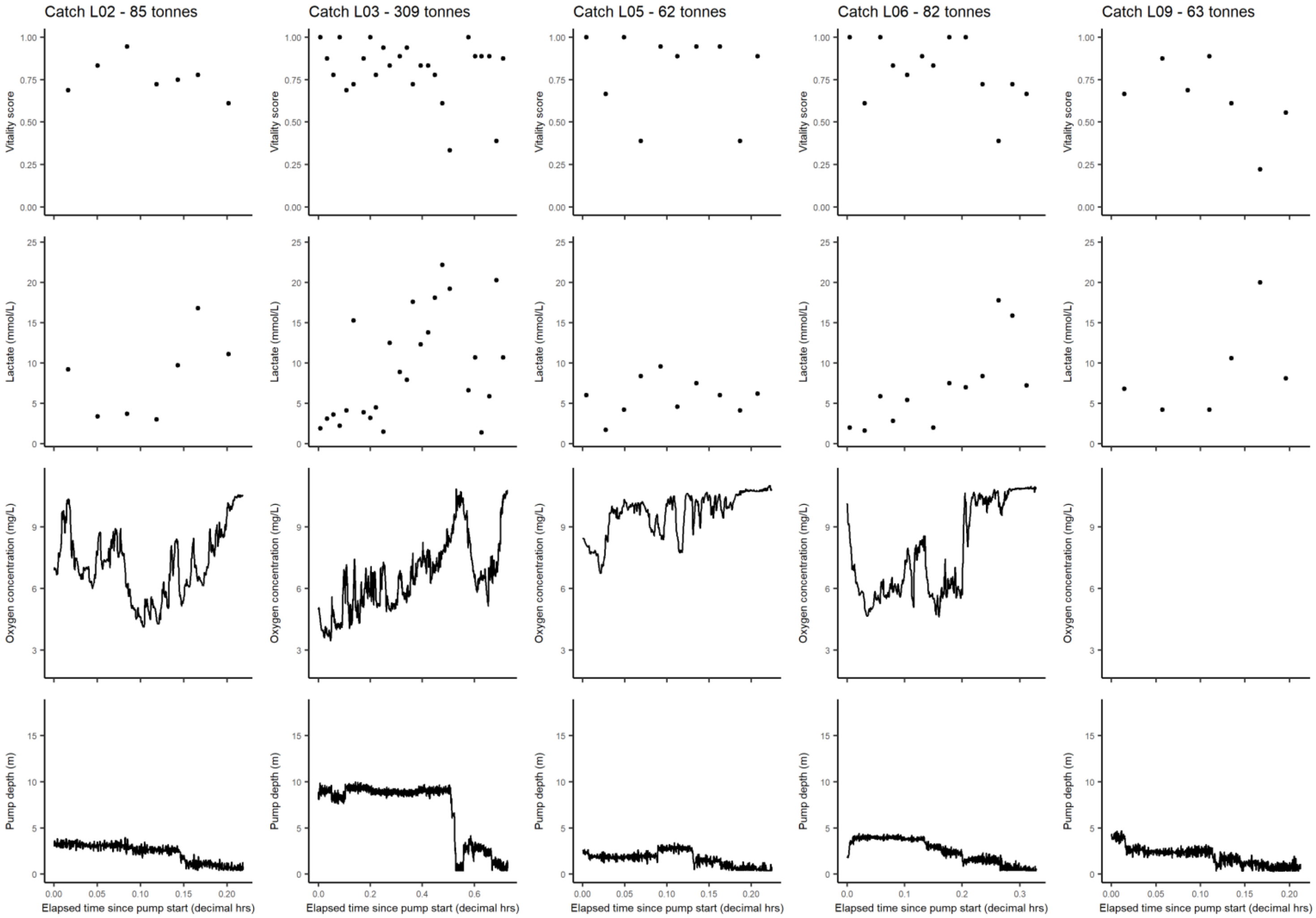


**Figure S5b**: Temporal change in pump depth and dissolved oxygen concentration (lower two panels) inside commercial purse seine catches of Atlantic mackerel (*Scomber scombrus*) during Trip 2 (refer to Table 5 in main article for further detail). Blood lactate and vitality scores from individual fish sampled throughout the pumping related crowding is also displayed (top two panels).

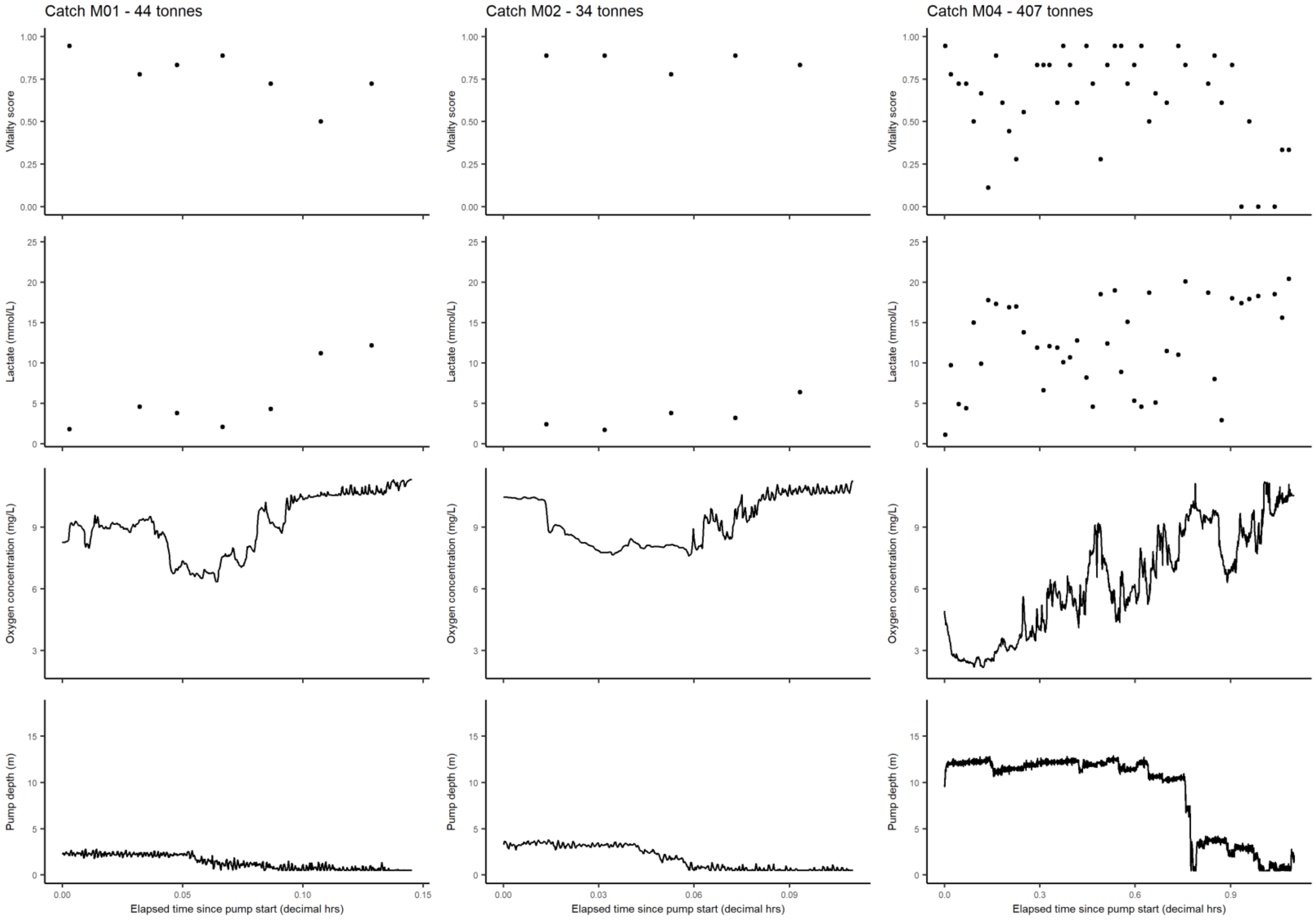


**Figure S5c**: Temporal change in pump depth and dissolved oxygen concentration (lower two panels) inside commercial purse seine catches of Atlantic mackerel (*Scomber scombrus*) during Trip 3 (refer to Table 5 in main article for further detail). Blood lactate and vitality scores from individual fish sampled throughout the pumping related crowding is also displayed (top two panels).

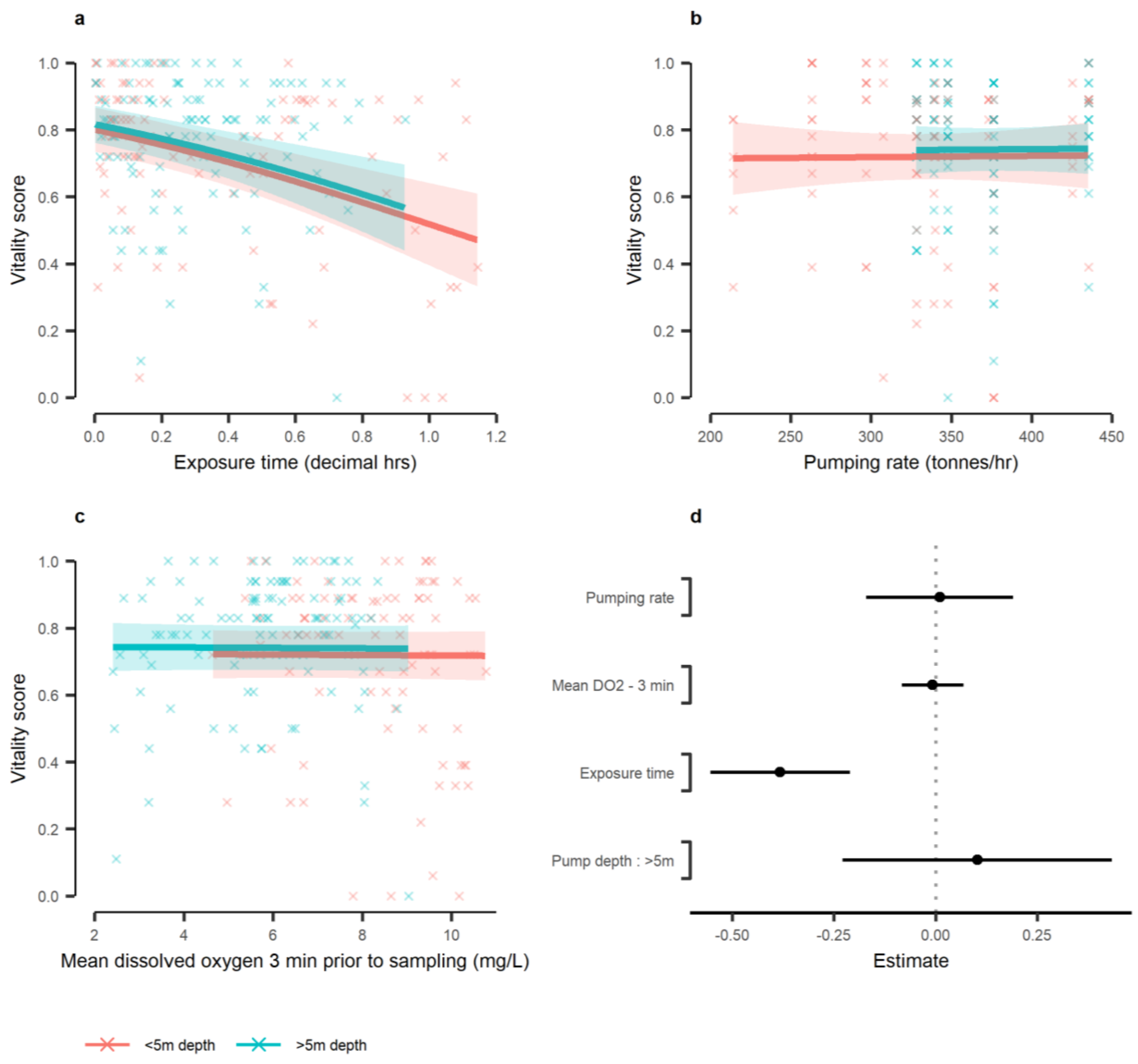


**Figure S6**: The model derived relationship between vitality scores and various drivers during crowding-related pumping of Atlantic mackerel (*Scomber scombrus*) during commercial purse seine capture. The model fits were constructed from averaging of three top competing models selected by Akaike information criterion. Fish were collected throughout crowding (after being pumped onboard) and assessed for behavioural vitality. **a**: the relationship with crowding exposure time, for the mean pumping rate (351 tonnes/hr) and mean dissolved oxygen ($DO_2$) in the net during the previous 3 mins prior to vitality sampling (6.90 mg/L). **b:** the relationship with pumping rate, for the mean exposure time (0.33 hrs) and mean $DO_2$. **c**: the relationship with $DO_2$ in the net during the previous 3 mins prior to vitality sampling, for the mean exposure time and mean pumping rate. The shaded area indicates the model derived 95% confidence interval, with the underlying data indicated as crosses. All relationships are factorised according to pump depth (either <5m depth or >5m depth). **d**: model coefficients (black points) for the averaged model, with 95% confidence intervals as whiskers. The dotted grey line indicates zero effect. All continuous model covariates were scaled prior to fitting.

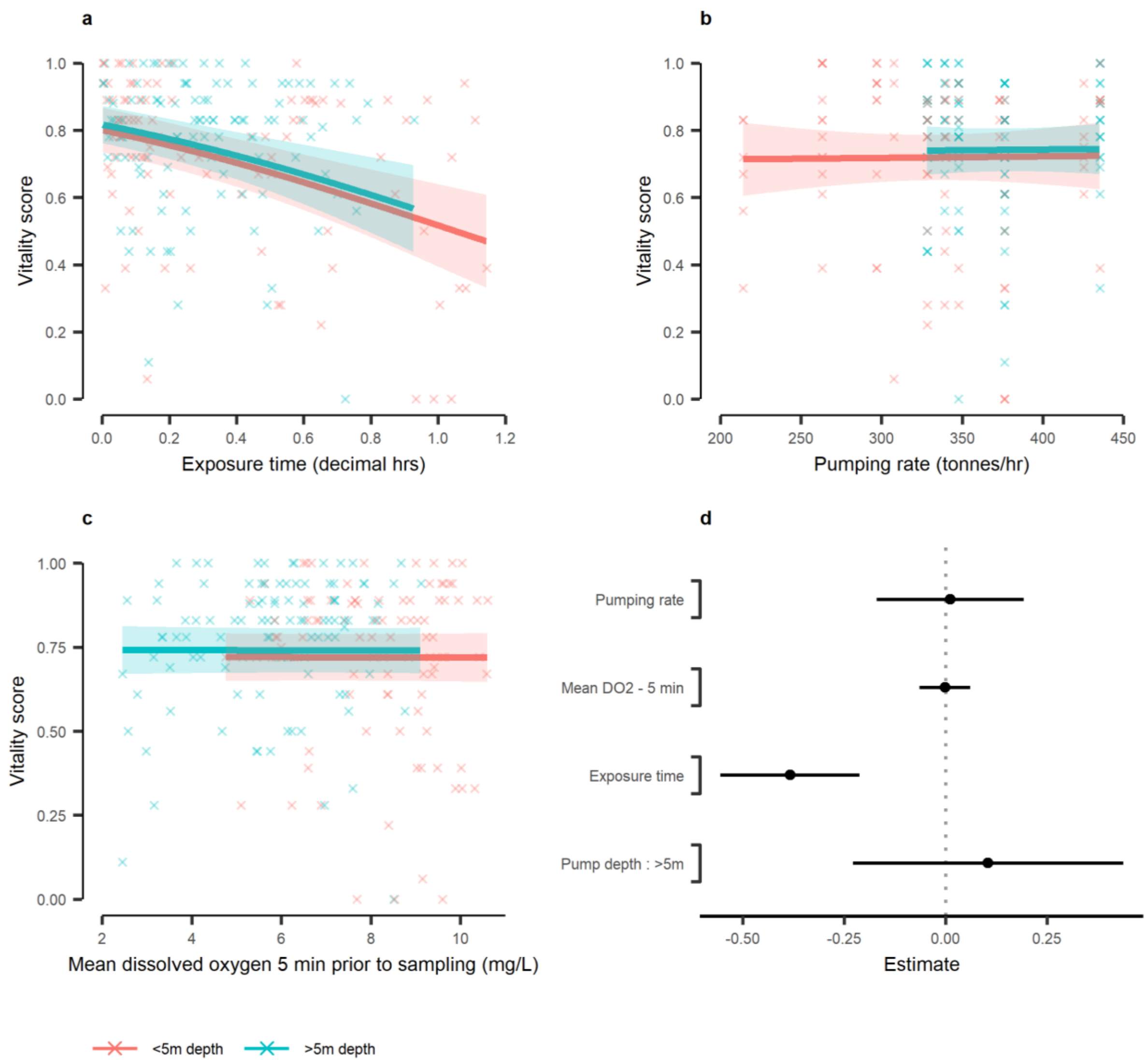


**Figure S7**: The model derived relationship between vitality scores and various drivers during crowding-related pumping of Atlantic mackerel (*Scomber scombrus*) during commercial purse seine capture. The model fits were constructed from averaging of three top competing models selected by Akaike information criterion. Fish were collected throughout crowding (after being pumped onboard) and assessed for behavioural vitality. **a**: the relationship with crowding exposure time, for the mean pumping rate (351 tonnes/hr) and mean dissolved oxygen ($DO_2$) in the net during the previous 5 mins prior to vitality sampling (6.95 mg/L). **b:** the relationship with pumping rate, for the mean exposure time (0.33 hrs) and mean $DO_2$. **c**: the relationship with $DO_2$ in the net during the previous 5 mins prior to vitality sampling, for the mean exposure time and mean pumping rate. The shaded area indicates the model derived 95% confidence interval, with the underlying data indicated as crosses. All relationships are factorised according to pump depth (either <5m depth or >5m depth). **d**: model coefficients (black points) for the averaged model, with 95% confidence intervals as whiskers. The dotted grey line indicates zero effect. All continuous model covariates were scaled prior to fitting.
